# Tissue Factor Pathway Inhibitor 2 Enhances Hepatocellular Carcinoma Chemosensitivity by Activating CCAR2-GADD45A-Mediated DNA Damage Repair

**DOI:** 10.7150/ijbs.111142

**Published:** 2025-07-11

**Authors:** Hongzhong Zhou, Liwen Zhu, Yajun Zhang, Lichan Chen, Dong-Mei Gou, Haohua Zhang, Rongmao Hua, Jianning Song, Chuanghua Qiu, Fu-Wen Yao, Xiafei Wei, Dayong Gu, Yong Xu

**Affiliations:** 1 Department of Laboratory Medicine, Shenzhen Institute of Translational Medicine, The First Affiliated Hospital of Shenzhen University, Shenzhen Second People's Hospital, Medical Innovation Technology Transformation Center of Shenzhen Second People's Hospital, Shenzhen University, China.; 2 Shenzhen Third People's Hospital, Southern University of Science and Technology, Shenzhen, China.; 3 Guangzhou University of Chinese Medicine, Guangzhou, China.; 4 Leland Stanford Junior University, USA.; 5 Key Laboratory of Gastrointestinal Cancer (Fujian Medical University), Ministry of Education, Fujian Medical University, China.; 6 Guangdong Provincial Clinical Research Center for Laboratory Medicine, China.

**Keywords:** hepatocellular carcinoma, chemoresistance, organoid, TFPI2, mRNA stability

## Abstract

Chemotherapy resistance presents a major challenge in the treatment of hepatocellular carcinoma (HCC), with the underlying molecular mechanisms largely unknown. This study aimed to investigate the role of tissue factor pathway inhibitor 2 (TFPI2) in modulating HCC chemosensitivity. We explored the impact of TFPI2 on sorafenib sensitivity in patient-derived organoids and mouse models using immunofluorescence analysis, chromatin immunoprecipitation, and RNA immunoprecipitation. We observed the downregulation of TFPI2 in HCC, and its deletion in mice (TFPI2^HKO^) accelerated DEN-induced liver tumorigenesis. Notably, TFPI2 overexpression increased sorafenib sensitivity in HCC organoids and *in vivo* models. Mechanistic insights indicated that TFPI2 stabilizes the mRNA of growth arrest and DNA damage-inducible alpha (GADD45A) by engaging the cell cycle and apoptosis regulator 2 (CCAR2), promoting GADD45A-mediated DNA damage and inhibiting homologous recombination repair. Furthermore, TFPI2 protects CCAR2 from ubiquitination-induced degradation by associating with the deubiquitinating enzyme BRCC3. We identified polydatin, a resveratrol glycoside, which upregulates TFPI2 and synergistically enhances the chemosensitizing effect of sorafenib in organoids and *in vivo*. TFPI2 plays a critical role in CCAR2-GADD45A-induced DNA damage repair, providing a strategy to enhance HCC chemosensitivity. Our findings elucidate the molecular intricacies of chemoresistance in HCC and reveal a potential therapeutic target for alleviating this resistance.

## Introduction

Liver cancer is a significant global health challenge with an increasing incidence. It is estimated that over 1 million individuals will be affected worldwide by 2025 1, 2. Radiotherapy and chemotherapy are primarily used to curb tumor progression 3. Sorafenib is the first molecular-targeted drug approved in several countries for first-line treatment of advanced liver cancer 4. However, only one-third of patients with advanced-stage HCC respond to sorafenib treatment, and many relapse within six months due to the high heterogeneity of HCC and changes in the tumor microenvironment 5. The main challenges in treating advanced HCC include the brief efficacy of sorafenib and the development of resistance 6.

Coagulation proteins, especially tissue factor pathway inhibitors (TFPI), are major inhibitors of the tissue factor (TF)-dependent coagulation pathway and play a role in tumor proliferation and metastasis. TFPI2, a second inhibitor of the TF-dependent pathway, is a Kunitz-type protease inhibitor that suppresses the activity of various proteases. The potential of TFPI2 as a tumor suppressor has been suggested in a variety of studies, as TFPI2 has been shown to inhibit extracellular matrix degradation in various malignancies, including pancreatic cancer 7, 8, prostate cancer 9, breast cancer 10, gastric cancer 11, glioblastoma 12, and HCC 13. In contrast, TFPI2 was found to be upregulated in glioblastoma 14, melanoma 15, ovarian clear cell carcinoma 16, and colorectal cancer 17. The expression heterogeneity of TFPI2 across various malignancies stems from three fundamental regulatory mechanisms that collectively determine its tumor-specific expression profiles. First, epigenetic modifications, particularly promoter hypermethylation 18 and repressive histone marks (e.g., H3K27me3 19, 20), mediate transcriptional silencing in multiple cancer types, as demonstrated in colorectal and gastric carcinomas. Second, transcription factor networks differentially regulate TFPI2 expression, while c-JUN/SP1/JNK activate its transcription in glioma 18, and TWIST1 recruits DNA methyltransferases for epigenetic silencing in ovarian malignancies. Third, dynamic tumor microenvironmental cues, including hypoxia-induced HIF-1α stabilization and inflammation-triggered IL-6/JAK/STAT signaling, create tissue-specific repression patterns. These multilayered regulatory mechanisms explain the observed tumor-type-dependent variation in TFPI2 expression. Meanwhile, emerging evidence has established TFPI2 as a positive regulator of tumor chemosensitivity across multiple cancer types. In nasopharyngeal carcinoma (NPC) cells, TFPI2 overexpression and a combination of cisplatin treatment were found to elicit a synergistic effect on promoting apoptosis and antitumor activity compared to cisplatin or TFPI2 treatment alone. This synergistic interaction highlights TFPI2's therapeutic potential as a chemosensitization target 21. However, the potential role of TFPI2 in modulating HCC drug sensitivity remains poorly characterized, representing a critical knowledge gap in liver cancer therapeutics.

GADD45A is a stress-inducible protein with increased expression under growth arrest conditions and after treatment with DNA-damaging agents. GADD45A, as a core regulator of the DNA damage response, can affect chemosensitivity in cancer through multiple mechanisms 22, such as increasing the effect of DNA-damaging drugs through inhibition of the CDK1/2-cyclin B complex that induces G2/M phase arrest in breast cancer 23; sustained activation of the JNK/p38/MAPK pathway reinforces genotoxic stress-induced apoptosis 24, 25. Recent studies have shown that cisplatin exerts its anti-HCC effects by upregulating GADD45A expression, contributing to enhanced chemotherapeutic efficacy 26, but the precise molecular mechanisms by which GADD45A enhances chemosensitivity in hepatocellular carcinoma (HCC) remain incompletely understood.

CCAR2 is a cell cycle and apoptosis regulator that significantly enhances the sensitivity of tumor chemotherapy through multiple molecular mechanisms. Following DNA damage, CCAR2 is phosphorylated by the apical kinases ATM/ATR. This phosphorylation enhances CCAR2's binding to SIRT1, leading to SIRT1 inhibition, subsequent p53 acetylation, and ultimately p53-dependent apoptosis 27, 28. Additionally, a study in non-small cell lung cancer demonstrated that high CCAR2 expression was positively correlated with the efficacy of cisplatin, further suggesting that CCAR2 has a chemosensitizing function as a new target for overcoming chemoresistance in HCC 27. However, the specific molecular mechanism by which CCAR2 regulates chemosensitization in hepatocellular carcinoma has not been fully elucidated.

In this study, our findings, for the first time, demonstrate that TFPI2 exerts a crucial role in the CCAR2-GADD45A-inducing DNA damage pathway, bringing groundbreaking new insights for alleviating the challenges posed by the development of HCC and chemotherapy resistance, heralding a new era of precision medicine and personalized therapeutic strategies.

## Methods

### Patients and tissue samples

Tumor samples and their adjacent normal counterparts were obtained from Shenzhen Second People's Hospital. All tissue samples were pathologically validated by pathologists and stored at -80 ℃ until examination. This study was approved by the Ethics Committee on Scientific Research of Shenzhen Second People's Hospital (protocol code: 2023-262-01, date of approval: 14 November 2023). All patients provided written informed consent following the guidelines of the 1964 Declaration of Helsinki and its subsequent revisions.

### Cell culture

HCC cell lines (MHCC97H, Huh-7, SNU449, and HLE) used in this study were purchased from Jennio Biotech Co., Ltd. (Guangzhou, China). The cells were cultured in Dulbecco's Modified Eagle Medium (DMEM), supplemented with 10% fetal bovine serum (FBS; Gibco), 100 U/mL penicillin, and 100 μg/mL streptomycin, followed by incubation at 37 °C under 5% CO_2_ in a cell incubator. The cells were cultured to 70-80% density and passaged. The cell lines were regularly authenticated using short tandem repeat analysis and monitored for mycoplasma contamination.

### CCK-8 assay

Briefly, 1 × 10^4^ cells/100 μL DMEM were cultured in each well of a 96-well plate and treated with different concentrations of sorafenib (0, 2, 4, 6, 8, 10 and 12 μM) for 24 h after cell attachment. Subsequently, 10 μL CCK8 solution was added to each well and incubated at 37 °C for 2 h. The viability of HCC cells was determined using a Cell Counting Kit 8 (HY-K0301; MCE, Shanghai, China) and measured at 450 nm using the BioTek Gen5 system (BioTek, USA). The viability of cells in each well was normalized to that of the control.

### DNA damage assay

Huh-7 or MHCC97H cells were seeded in a 24-well plate (1.0 × 10^5^ cells/well) for 24 h and treated with 10 μM sorafenib for 12 h. The cells were washed with cold PBS and fixed for 20 min at room temperature. Non-specific binding sites were blocked at room temperature for 20 min using QuickBlock™ Blocking Buffer for Immunostaining. The cells were incubated with anti-γ-H2AX, anti-RAD51, or anti-53BP1 primary antibody overnight at 4 °C. After washing three times for 5 min each, the cells were incubated with a fluorescein isothiocyanate (FITC)-conjugated secondary antibody at room temperature for 1 h and then stained with DAPI for 10 min. Specimens were examined and imaged using a ZEISS LSM 800 confocal microscope (Zeiss, Germany).

### RNA fluorescence *in situ* hybridization (FISH)

Cells in the logarithmic growth phase were seeded in a 48-well plate and allowed to fully adhere for 24 h. The excess medium was washed three times with PBS, fixed in 4% paraformaldehyde for 15 min at room temperature, and permeabilized with 0.5% Triton X-100 for 30 min. After blocking the non-specific binding sites with the blocking solution in the RNA Fluorescent *In Situ* Hybridization Kit (Genepharma, China), the denatured probe was mixed with incubation buffer, and the cells were incubated overnight in a 37 °C incubator. After washing away excess non-specific binding, cells were incubated with the diluted primary antibody overnight at 4 °C. On the next day, cells were incubated with secondary antibodies at room temperature, and the cell nuclei were stained with DAPI and photographed using a confocal microscope (ZEISS, Germany). mRNA probes used to identify the GADD45A mRNA were synthesized by GenePharma, China. The antibodies used in this assay are listed in **Table S4**.

### Animal studies

TFPI2^flox/flox^ mice and Alb-iCre mice on a C57/6J background were purchased from GemPharmatech (Nanjing, China). Hepatocyte-specific TFPI2 gene knockout mice (TFPI2^flox/flox^ Alb-iCre, TFPI2^HKO^) and their littermates (TFPI2^flox/flox^) were generated by crossing TFPI2^flox/flox^ mice with Alb-iCre mice. The genotypes of the transgenic mice were determined by polymerase chain reaction (PCR) of mouse tail DNA. To establish the xenograft tumor model, cells were inoculated subcutaneously into the thigh of nude mice at a density of 3 × 10^6^ cells and an injection volume of 150 μL. Tumor volumes were measured weekly according to the formula V = (L × W^2^) / 2, where L and W are the length and width of the tumor. For drug treatment, mice bearing subcutaneous tumors were randomly divided into different groups and treated with drugs or the negative control (DMSO) according to the group assignment. Polydatin (100 mg/kg) and sorafenib (30 mg/kg) were administered on alternate days by gavage. Dosing was continued for two weeks, and tumor volume was measured before each dose. In the orthotopic tumor model, 10^6^ cells in 20 μL DMEM mixed with 20 μL Matrigel solution (Corning, USA) were injected into the left liver lobe of the nude mice. The mice were euthanized, and the tumors were fixed in formalin and embedded in paraffin. For each tumor tissue block, consecutive sections were prepared and subjected to hematoxylin and eosin (H&E) and IHC staining. All animal experiments were approved by the Shenzhen Second People's Hospital (protocol code: 202300208, date of approval: 15 November 2023), and all procedures were performed under institutional guidelines. All animal tumors have a diameter not exceeding 20 mm.

### Randomized grouping of animal experiments and inclusion and exclusion criteria

Tumor volume-stratified randomization was performed using computer software to ensure balanced group allocation. Unaware of treatment groups, blinded researchers conducted measurements following standardized protocols, with independent data collection to maintain objectivity. Animals exhibiting >20% weight loss or technical death were excluded from the analysis.

### Organoid viability assay

Briefly, organoids were digested into single cells, seeded in 96-well plates, and treated with the indicated gradient drugs (sorafenib and polydatin) for 6 days. Observed and photographed organoid images under a microscope. Subsequently, cell viability was determined using the Cell Counting-Lite 3D Luminescent Cell Viability Assay Kit (Vazyme, China) according to the manufacturer's instructions. Fresh medium containing the drug was replaced daily in the polydatin group but not the sorafenib group. All experiments were performed in triplicate.

### Statistical methodology

Statistical analyses were performed using GraphPad Prism V9 (GraphPad Software, Inc., La Jolla, CA, USA). Unless otherwise stated, the data are shown as the mean ± SEM of n = 3 biological replicates. An unpaired two-tailed Student's t-test was performed to compare the differences in cell viability, gene expression between different groups, and tumor volume. A paired two-tailed Student's t-test was used to compare TFPI2 expression between HCC tissues and paired adjacent tissues. The chi-square test was used to compare the association between TFPI2 expression and the clinicopathological parameters of patients with HCC. Kaplan-Meier curves and log-rank tests were used to analyze TFPI2 expression and the survival of patients with HCC. A p-value of < 0.05 was deemed statistically significant.

Additional methods are provided in Supplementary Methods.

## Results

### Patients with HCC with low TFPI2 expression had a poorer prognosis

**Figure 1:**
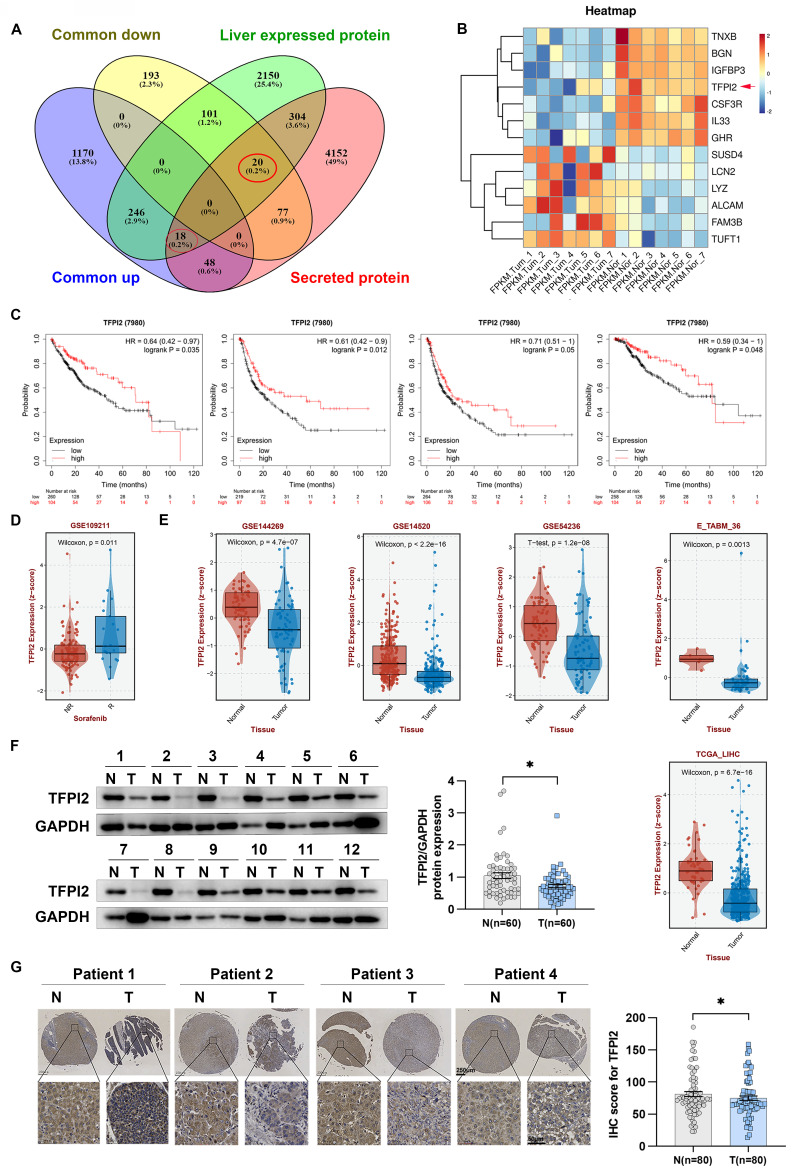
** TFPI2 is downregulated in HCC.** (A) Venn diagrams show 1,482 commonly upregulated and 391 downregulated genes from TCGA and seven paired RNA-seq from our lab (|FC| ≥ 2, *p* < 0.05). We analyzed 4,619 genes encoding secreted proteins and 2,839 liver-expressed genes, identifying 18 upregulated and 20 downregulated candidates. (B) Heatmap illustrating expression profiles of 13 potential secreted proteins from 7 HCC patient-derived samples. (C) Kaplan-Meier curves for OS (*p* = 0.035), RFS (*p* = 0.012), PFS (*p* = 0.05), and DSS (*p* = 0.048) based on TFPI2 expression, analyzed by the log-rank test. (D) GSE109211 dataset examines TFPI2 response to sorafenib; NR: No Response; R: Response. (E) Other datasets (GSE144269, GSE14520, GSE54236, E_TABM_36, TCGA_LIHC) analyze TFPI2 expression. (F) Representative western blotting shows TFPI2 expression in 60 paired HCC tissues. Data are presented as mean ± SEM by Student's t-test. *p < 0.05. (G) Representative IHC images show TFPI2 expression in paired tissue from tissue microarrays of HCC patients. IHC staining was evaluated using histochemical scoring. Data are presented as mean ± SEM. *p < 0.05. Scale bars: 250 µm (LM), 50 µm (HM).

Secreted proteins play an essential role in medicine and biology as potential therapeutic targets and diagnostic markers. However, their specific role in HCC development is not well understood. To explore this, we analyzed gene expression profiles from the TCGA-LIHC database and RNA-seq data from seven patients, identifying 1,482 upregulated and 391 downregulated genes. We then examined 4,619 genes encoding secreted proteins and 2,839 liver-expressed genes, finding 18 upregulated and 20 downregulated genes (**Figure 1A**). Using QIAGEN Ingenuity Pathway Analysis, we found that seven downregulated and six upregulated genes were significantly enriched in regulating organismal injury and abnormalities, developmental disorders, and inflammatory response (**Figure 1B and Figure S1A**). Survival analysis showed that dysregulated expression of TFPI2, TNXB, IL33, IGFBP3, and GHR correlated with overall survival, relapse-free survival, progression-free survival, and disease-specific survival (**Figure 1C** and **Figure S1B**). Notably, TFPI2 and TNXB expression levels were higher in patients who responded to sorafenib and lower in those who did not respond; IL33 and IGFBP3 did not show these distinct expression patterns, and GHR levels exhibited an inverse correlation with the response to sorafenib (**Figure 1D** and **Figure S1C**). These findings suggested that TFPI2 and TNXB could be associated with chemotherapeutic drug sensitivity. To confirm TFPI2 and TNXB expression levels in HCC, we analyzed their clinical significance using the GSE144269, GSE14520, GSE54236, E_TABM_36, and TCGA_LIHC datasets. TFPI2 expression was markedly reduced in the five datasets (GES144269, *p* = 4.7e-07; GSE14520, *p* < 2.2e-16; GES54236, *p* = 1.2e-08; E_TABM_36, *p* = 0.0013; TCGA-LIHC, *p* = 6.7e-16; **Figure 1E**), whereas TNXB expression was downregulated only in GSE144269 (*p* = 1.9e-13) and TCGA-LIHC (*p* = 6.9e-10) but not in the other three databases (**Figure S1D**). Therefore, we focused on TFPI2 for further investigation. Next, we evaluated TFPI2 expression in our HCC cohort and found that TFPI2 expression was significantly lower in 60 patients with HCC (**Figure 1F and Figure S1E**). The low expression of TFPI2 was inversely correlated with AFP (*p* < 0.05) and tumor size (*p* < 0.05), while no correlation was found with other clinicopathological parameters (**Table S1**). Upon examining the IHC array, TFPI2 expression in HCC tissues was reduced compared to that in the para-cancerous tissues (**Figure 1G**), but the mechanism of its downregulation remains unknown. It has been reported that the promoter of TFPI2 is regulated by acetylation transcription 29. Since DDX17 is a significant transcription factor in liver cancer, we speculated whether DDX17 can regulate TFPI2 expression. Results showed that DDX17 overexpression significantly reduced both the mRNA and protein levels of TFPI2 in HCC cells, while DDX17 silencing upregulated TFPI2 expression (**Figure S2A-D**). Mechanistically, dual-luciferase reporter assays revealed that DDX17 overexpression suppressed the transcriptional activity of the TFPI2 promoter, whereas DDX17 knockdown enhanced it (**Figure S2E**). Chromatin immunoprecipitation (ChIP)-PCR further confirmed the direct binding of DDX17 to the TFPI2 promoter region (**Figure S2F**), suggesting that DDX17 inhibits TFPI2 transcription by directly targeting its promoter. Collectively, the data suggested that the downregulation of TFPI2 expression could be linked to the malignant development of HCC.

### TFPI2 suppresses the malignant proliferation of HCC *in vitro* and *in vivo*

**Figure 2:**
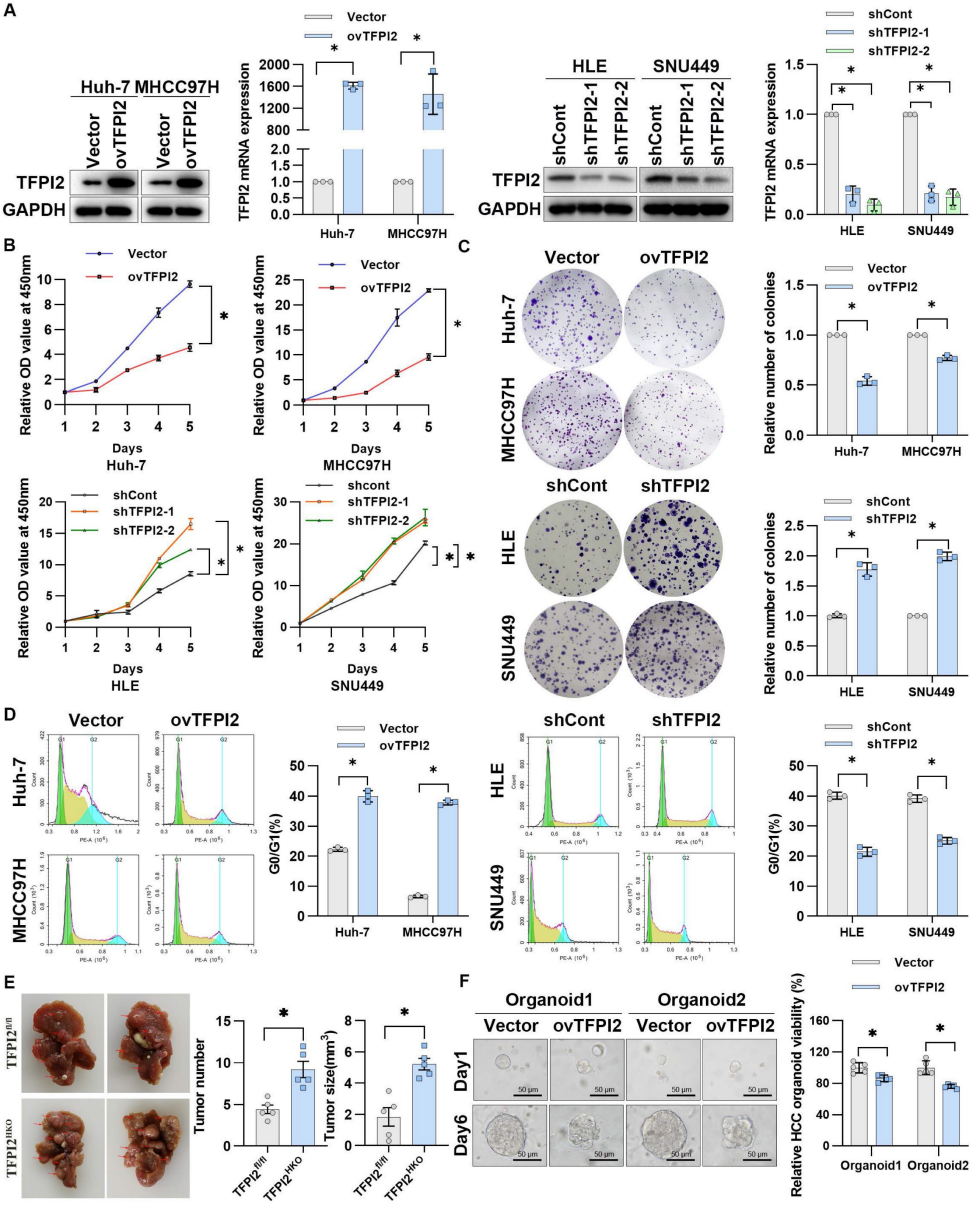
**TFPI2 suppresses HCC proliferation both *in vitro* and *in vivo***. (A) Western blotting and RT-qPCR verify TFPI2 overexpression in Huh-7 and MHCC97H cells and knockdown in HLE and SNU449 cells. Analyze the grayscale values with ImageJ; data are shown as the mean ± SEM. *p < 0.05*.* (B) CCK8 assay assessed the effect of TFPI2 overexpression or knockdown on HCC cell proliferation. *p < 0.05. (C) Colony formation assay examines the impact of TFPI2 overexpression or knockdown on HCC colony formation. Count cell colonies with ImageJ; data are shown as the mean ± SEM. *p < 0.05*.* (D) Flow cytometry detects the effect of TFPI2 overexpression or knockdown on the cell cycle. Data are shown as the mean ± SEM. *p < 0.05. (E) Representative liver images and tumor numbers from 10-month-old DEN-treated TFPI2^fl/fl^ and TFPI2^HKO^ mice. Data are shown as the mean ± SEM. *p < 0.05*.* (F) Organoid models assessed the impact of TFPI2 overexpression on organoid growth. Representative organoid images were shown. Organoid viability was measured by the 3D Cell Viability Assay Kit and presented as a histogram. Data are shown as the mean ± SEM. *p < 0.05*.* Scale bar: 50 µm.

To examine the effect of TFPI2 on HCC, we first detected the expression of TFPI2 in different liver cancer cells (**Figure S3A**). We then generated stable TFPI2-overexpression and TFPI2-knockdown HCC cells by lentiviral transduction based on TFPI2 expression levels (**Figure 2A**). The CCK8 assay showed that TFPI2 overexpression significantly inhibited the viability of Huh-7 and MHCC97H cells, whereas TFPI2 knockdown increased it (**Figure 2B**). Likewise, the colony formation assay revealed that TFPI2 overexpression reduced clonogenicity, whereas its downregulation enhanced it (**Figure 2C**). Cell cycle analysis revealed that TFPI2 overexpression caused G0/G1 phase arrest in HCC cells (**Figure 2D**). To assess the *in vivo* effects of TFPI2, hepatocyte-specific TFPI2 knockout mice (TFPI2^HKO^) were generated by crossing TFPI2^fl/fl^ mice with Alb-Cre mice. The expression of TFPI2 was markedly diminished in the liver tissues of TFPI2^HKO^ mice (**Figure S3B**). The occurrence of liver tumors was higher in TFPI2^HKO^ mice compared to TFPI2^fl/fl^ mice following a 10-month treatment with diethylnitrosamine (DEN)/CCl_4_ (**Figure 2E**). Additionally, we established orthotopic and subcutaneous xenograft tumor models using TFPI2-overexpressing HCC cells. The growth of TFPI2-overexpressing xenografts was slower than that of controls, as confirmed by H&E and IHC staining (**Figure S3C-F**). Importantly, patient-derived organoids (PDOs) showed that TFPI2 overexpression led to fewer and smaller organoids (**Figure 2F**). Collectively, these results indicated that TFPI2 could suppress HCC cell proliferation both *in vitro* and *in vivo*.

### TFPI2 mediates HCC growth inhibition by regulating the GADD45A-STAT3 pathway

**Figure 3:**
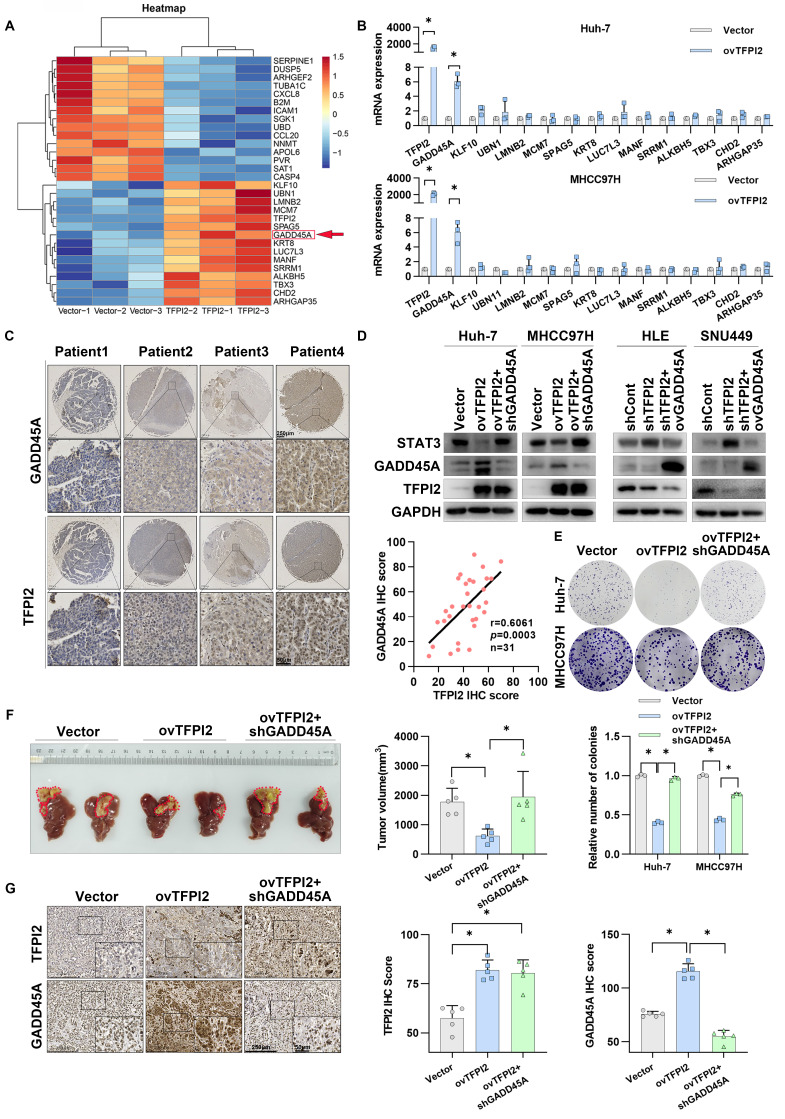
**TFPI2 inhibits HCC growth by regulating the GADD45A-STAT3 pathway.** (A) Heatmap shows differential gene expression in TFPI2-overexpressed and control MHCC97H cells. (B) RT-qPCR validates gene expression after TFPI2 overexpression. Data are shown as the mean ± SEM of n = 3 biological replicates. (C) IHC detects TFPI2 and GADD45A expression in 31 cases of HCC tissue microarray. Pearson analysis assesses their correlation, *p* = 0.0003. Scale bar: 250 µm (LM), 50 µm (HM). (D) Western blotting determines the effect of GADD45A knockdown in TFPI2-overexpressing cells or GADD45A upregulation in TFPI2-knockdown cells on the GADD45A-STAT3 pathway. (E) Colony formation detects the effect of GADD45A knockdown in TFPI2-overexpressing HCC cells. Count cell colonies with ImageJ; data are shown as the mean ± SEM. *p < 0.05*.* (F) Orthotopic tumor model was established using TFPI2-overexpressing MHCC97H cells with GADD45A knockdown, and tumor volume was measured after 4 weeks. TFPI2 overexpression inhibits liver tumor volume, while GADD45A knockdown reverses this effect. (G) IHC examines TFPI2 and GADD45A expression in harvested tumor tissues. IHC staining was evaluated using histochemical scoring. Data are presented as mean ± SEM. *p < 0.05. Scale bar: 250 µm (LM), 50 µm (HM).

To investigate the mechanism of TFPI2 on HCC proliferation, we conducted RNA-seq analysis on TFPI2-overexpressing and control MHCC97H cells, identifying the top 30 differentially expressed genes (**Figure 3A**). RT-qPCR validated these findings, with growth arrest and DNA-damage-inducible protein 45 alpha (GADD45A) mRNA levels being most significantly increased in TFPI2-overexpressing HCC cells (**Figure 3B** and **Figure S4A**). TFPI2 overexpression also upregulated GADD45A protein levels, while its knockdown reduced both mRNA and protein levels (**Figure S4B-C**). Kaplan-Meier analysis revealed that low GADD45A levels were associated with poor prognosis, consistent with TFPI2 expression (**Figure S4D**). IHC assay results confirmed a positive correlation between TFPI2 and GADD45A (**Figure 3C**). Given GADD45A's role in the STAT3 pathway 30, we investigated its involvement in TFPI2-mediated HCC proliferation. Rescue assays revealed that GADD45A knockdown reversed the TFPI2-induced downregulation of STAT3, while GADD45A overexpression counteracted the upregulation of STAT3 in TFPI2-knockdown cells (**Figure 3D**)**.** Furthermore, GADD45A knockdown partially reversed the inhibitory effect of TFPI2 on HCC cell proliferation. Conversely, the overexpression of GADD45A partially reversed the increased proliferation induced by TFPI2 knockdown (**Figure 3E** and **Figure S4E-F**). *In vivo*, TFPI2 overexpression decreased tumor volume in an orthotopic nude mouse liver cancer model, but GADD45A knockdown reversed this effect (**Figure 3F-G**). These findings suggest that TFPI2 suppresses HCC proliferation by activating the GADD45A-STAT3 pathway.

### TFPI2 increases the mRNA stability of GADD45A by binding to CCAR2

**Figure 4:**
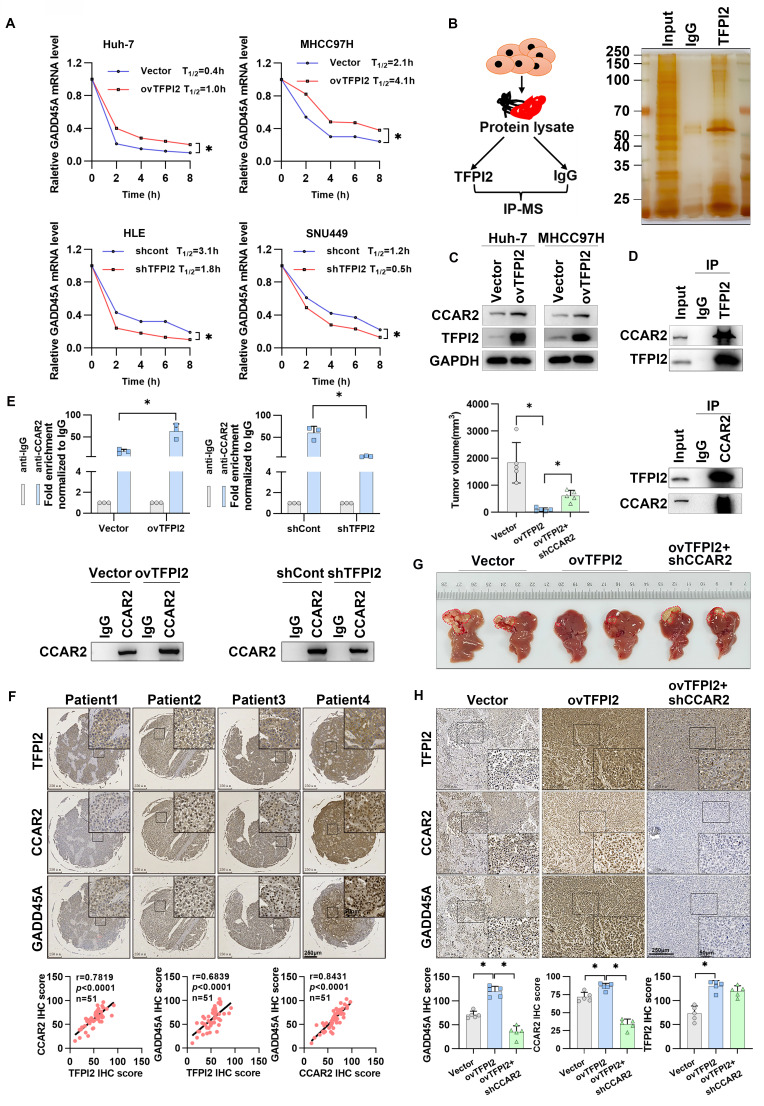
**TFPI2 increases GADD45A mRNA stability by binding to CCAR2.** (A) After treatment with Actinomycin D for 2,4,6,8h, GADD45A mRNA half-life was detected by RT-qPCR in HCC cells with TFPI2 overexpression or depletion. *p < 0.05. (B) The schematic shows TFPI2-binding protein screening by IP-MS.(C) Western blot was performed to detect the effect of TFPI2 overexpression on CCAR2. (D) IP experiments verified the TFPI2-CCAR2 interaction. (E) RIP experiment assesses the effect of TFPI2 on CCAR2 protein and GADD45A mRNA enrichment. *p < 0.05. (F) IHC staining detects TFPI2, CCAR2, and GADD45A proteins in HCC tissue microarray. Pearson analysis assesses their correlation. Scale bars: 250 µm (LM), 50 µm (HM). (G) TFPI2-overexpressing MHCC97H cells with CCAR2 knockdown were injected into the liver of mice, and tumor volume was measured after 4 weeks. (H) Expression of TFPI2, CCAR2, and GADD45A was detected by IHC. IHC staining was evaluated using histochemical scoring. Data are presented as mean ± SEM. *p < 0.05. Scale bars: 250 µm (LM), 50 µm (HM).

Given that TFPI2 expression upregulates both mRNA and protein levels of GADD45A (**Figure 3B** and **S4B**), we investigated whether this regulation occurs transcriptionally or translationally. We found that overexpression of TFPI2 did not affect the protein half-life of GADD45A while using inhibitors of protein degradation with different pathways (**Figure S5A**). Dual-luciferase assays indicated that the promoter activity of GADD45A was assayed after overexpression or knockdown of TFPI2, and the results did not show any significant changes, suggesting that TFPI2 did not affect GADD45A promoter activity (**Figure S5B**). Interestingly, GADD45A mRNA half-life increased from 0.4 to 1 h and 2.1 to 4.1 h in TFPI2-overexpressing cells, respectively, while it decreased from 3.1 to 1.8 h and 1.2 to 0.5 h in TFPI2-knockdown cells (**Figure 4A**). This suggests that TFPI2 enhances GADD45A expression by increasing mRNA stability.

Given that TFPI2 lacks RNA-binding domains, we hypothesized that TFPI2 may participate in RNA regulation by forming a complex with RNA-binding proteins (RBPs). Therefore, we performed IP and LC-MS to screen for RBPs capable of binding to TFPI2 (**Figure 4B**). LC-MS results identified 16 RBPs that may interact with TFPI2 (**Figure S5C-E**). Given our finding that TFPI2 upregulates and binds to the cell cycle and apoptosis regulator 2 (CCAR2) protein (**Figure 4C-D**) and GADD45A can be significantly upregulated by CCAR2 (**Figure S5F**), we focused on CCAR2. RIP and FISH experiments revealed that CCAR2 directly binds to GADD45A mRNA in HCC cells (**Figure S5G-H**). Importantly, TFPI2 overexpression increased CCAR2-GADD45A mRNA binding, while knockdown decreased it (**Figure 4E**). We further analyzed representative staining of these three proteins and found significant positive correlations between TFPI2, CCAR2, and GADD45A expression (**Figure 4F**). To investigate whether CCAR2 participates in the TFPI2-mediated GADD45A-STAT3 pathway, CCAR2 knockdown reversed the effects of TFPI2 overexpression on GADD45A and STAT3 expression. Conversely, CCAR2 overexpression counteracted the effects of TFPI2 knockdown, as well as the inhibition of HCC cell growth (**Figure S6A-F**). *In vivo*, TFPI2 overexpression decreased tumor volume in an orthotopic nude mouse liver cancer model, but CCAR2 knockdown reversed this effect (**Figure 4G-H**). Collectively, the above results suggest that TFPI2 enhances GADD45A mRNA stability by binding to CCAR2.

### TFPI2 binds with the deubiquitinating enzyme BRCC3 to prevent CCAR2 protein degradation

**Figure 5:**
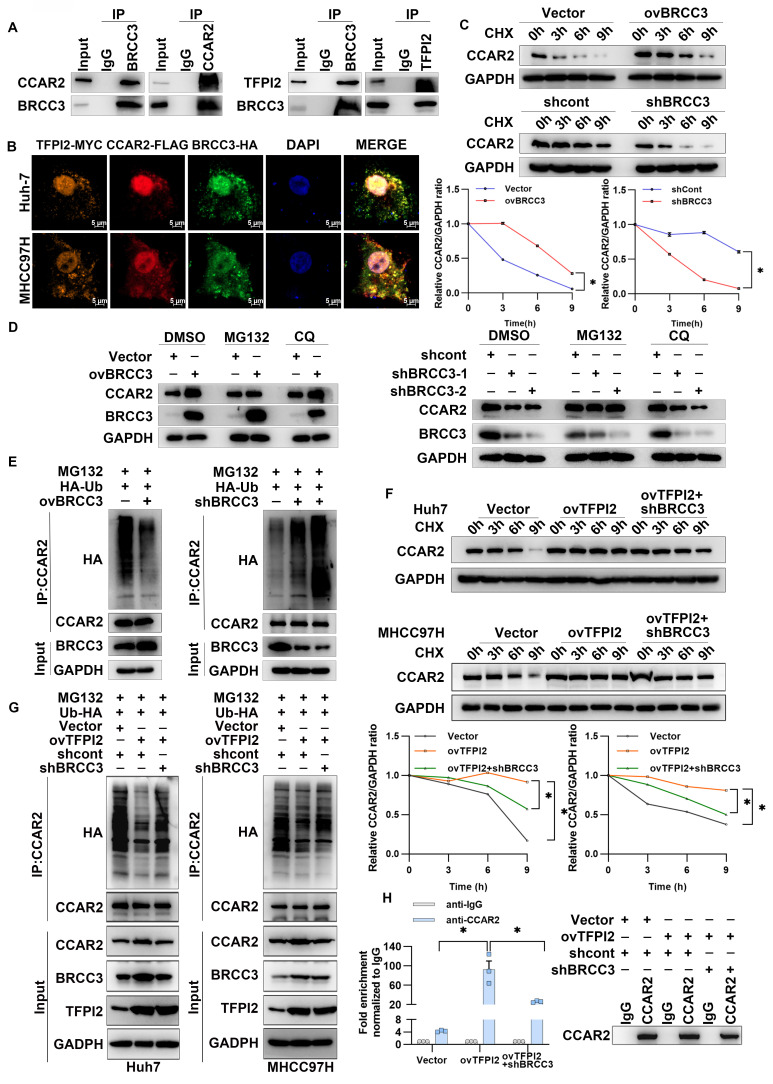
** TFPI2 binds with deubiquitinating enzyme BRCC3 to prevent CCAR2 protein degradation.** (A) IP experiments confirmed the interaction between BRCC3, TFPI2, and CCAR2. (B) IF assays determined the subcellular colocalization of TFPI2, BRCC3, and CCAR2. Scale bar = 5 μm. (C) HCC cells were transfected with a BRCC3 plasmid (2.5 µg) or BRCC3 shRNA (MOI=5), treated with cycloheximide (CHX) (300 µM), and collected at 0, 3, 6, and 9 h. Western blotting assay to detect the CCAR2 protein stability. Analyze the grayscale values with ImageJ; data are shown as the mean ± SEM. *p < 0.05. (D) HCC cells transfected with a BRCC3 plasmid (2.5 µg) or BRCC3 shRNA (MOI=5) were treated with or without the proteasome inhibitor MG132 (10 μM for 2 h) and the autophagy inhibitor chloroquine (CQ) (10 μM for 2 h) during CHX treatment. (E) BRCC3-overexpressing or BRCC3-knockdown cells co-transfected with HA-tagged ubiquitin (HA-Ub) plasmids (1.25 µg) were subjected to IP after being treated with 10 µM MG132 for 2 h. (F) HCC cells were transfected with BRCC3 shRNA in TFPI2-overexpressing cells, treated with CHX (300 µM), and collected at 0, 3, 6, and 9 h. Western blotting assay to detect the CCAR2 protein stability. Analyze the grayscale values with ImageJ; Data are shown as the mean ± SEM. *p < 0.05. (G) Following BRCC3 knockdown in TFPI2-overexpressing cells, IP was performed on MHCC97H cells co-transfected with HA-tagged ubiquitin (HA-Ub) plasmid after treatment with 10 μM MG132 for 2 h. (H) RIP experiment assesses the effect of BRCC3 knockdown on CCAR2 protein and GADD45A mRNA enrichment. Data are shown as the mean ± SEM. *p < 0.05.

To examine the mechanism through which TFPI2 regulates CCAR2, IP-MS results suggested that TFPI2 may interact with BRCC3, which was confirmed by IP assays (**Figure 4B and Figure 5A**). Additionally, immunofluorescence co-localization showed that TFPI2, CCAR2, and BRCC3 had clear co-localization in liver cancer cells (**Figure 5B**). Notably, BRCC3 overexpression increased CCAR2 protein levels without affecting mRNA levels, while BRCC3 knockdown had the opposite effect (**Figure S7A-D**), suggesting that BRCC3 did not affect CCAR2 transcription levels. Cycloheximide (CHX) treatment showed that CCAR2 protein degraded more slowly in BRCC3-overexpressing HCC cells, whereas BRCC3 knockdown accelerated degradation (**Figure 5C**). We then found the proteasome inhibitor MG132, but not the autophagosome pathway inhibitor chloroquine (CQ), reversed the effects of BRCC3 on CCAR2 protein levels (**Figure 5D**). Furthermore, BRCC3 overexpression reduced CCAR2 ubiquitination, while its knockdown increased it (**Figure 5E**). Critically, BRCC3 knockdown markedly reversed the TFPI2 overexpression-induced enhancement of CCAR2 protein stability and reduction in ubiquitination levels (**Figure 5F-G**). In parallel, BRCC3 depletion abrogated the TFPI2-driven stabilization of GADD45A mRNA in TFPI2 overexpressing cells (**Figure 5H**). Together, these findings establish that TFPI2 enhances CCAR2 protein stability through BRCC3-mediated deubiquitination, thereby elevating GADD45A expression levels.

### TFPI2 enhances HCC cell sensitivity toward sorafenib both *in vitro* and *in vivo*

**Figure 6:**
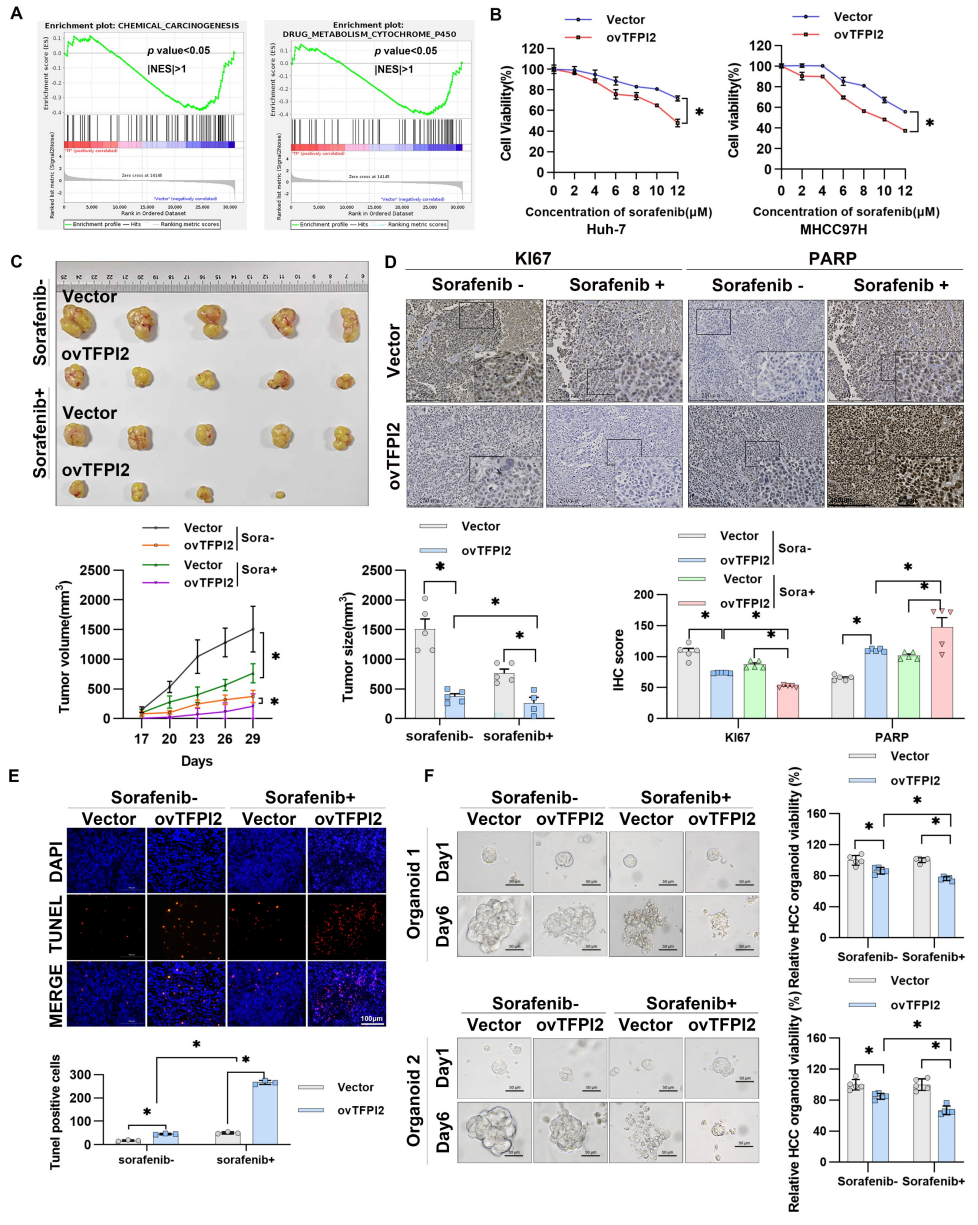
**TFPI2 enhances HCC sensitivity toward sorafenib *in vitro* and *in vivo.*** (A) RNA-seq was performed to sequence TFPI2-overexpressing cells, followed by GSEA enrichment analysis of related pathways. (B) A CCK8 assay measured HCC cell viability after TFPI2 overexpression and sorafenib treatment at various concentrations. Data are shown as the mean ± SEM of n = 3 biological replicates. *p < 0.05*.* (C) Mice were divided into sorafenib and vehicle groups after being injected with MHCC97H with TFPI2 overexpressing or control for two weeks. Sorafenib was administered at 30 mg/kg via gavage, with tumor volumes measured every other day. After two weeks, mice were sacrificed, and xenografts were harvested. Tumor volume and tumor size data are presented as mean ± SEM. *p < 0.05. (D) IHC detected Ki67 and PARP expression in tumor sections. IHC staining was evaluated using histochemical scoring. Data are presented as mean ± SEM. *p < 0.05. Scale bars: 250 µm (LM), 50 µm (HM). (E) TUNEL staining assessed apoptosis in tumor sections. TUNEL-positive cells were counted with ImageJ. Data are presented as mean ± SEM. *p < 0.05. Scale bar: 100 µm. (F) HCC organoids were generated to verify TFPI2 overexpression effects on sorafenib sensitivity, with sensitivity measured after one week using a 3D cell viability assay kit and presented as a histogram. Data are presented as mean ± SEM. *p < 0.05. Scale bar: 50 µm.

Gene Set Enrichment Analysis (GSEA) revealed that TFPI2 likely plays a pivotal role in regulating cellular response to environmental stressors, specifically in chemical carcinogenesis and drug metabolism involving cytochrome P450 (**Figure 6A**). Moreover, TFPI2 expression level positively correlated with the sensitivity of sorafenib response in patients with HCC (**Figure 1D**), indicating a potential association between TFPI2 and chemotherapeutic drug sensitivity. To investigate TFPI2's role in sorafenib resistance, we generated sorafenib-resistant HCC cell lines and observed a marked reduction in TFPI2 expression compared to sorafenib-sensitive counterparts (**Figure S8A-B**). Functional studies revealed that TFPI2 overexpression significantly improved the sensitivity of HCC cells to sorafenib, while its knockdown had the opposite effect (**Figure 6B** and **Figure S8C**). Notably, TFPI2 modulation similarly influenced responses to other inhibitors (regorafenib and varlitinib), indicating a broader role in HCC chemosensitivity (**Figure S8D**). Colony formation assays demonstrated that TFPI2 overexpression synergized with sorafenib to suppress clonogenic survival (**Figure S8E**). Sorafenib-induced apoptosis was enhanced by TFPI2 overexpression (**Figure S8F**). Furthermore, TFPI2 overexpression under sorafenib treatment significantly upregulated BRCC3, CCAR2, and GADD45A protein levels **(Figure S8G)**, suggesting that TFPI2 enhanced the sensitivity of HCC to sorafenib through the BRCC3/CCAR2/GADD45A axis. *In vivo*, TFPI2 overexpression significantly reduced tumor volume and size in sorafenib-treated mice (**Figure 6C**), consistent with a higher proportion of PARP-positive and TUNEL-positive cells and lower Ki67 expression (**Figure 6D-E**), indicating enhanced sorafenib-induced apoptosis in TFPI2-overexpressing cells. Additionally, PDOs showed that TFPI2-overexpressing HCC PDOs were more sensitive to sorafenib than controls (**Figure 6F**). To compare organoids and clinical relevance, we correlated organoid-based sorafenib sensitivity profiles with clinical outcomes in five HCC patients who experienced disease progression during sorafenib therapy. Among them, organoid models from four individuals exhibited localized drug resistance patterns, while one patient's organoids maintained homogeneous sensitivity across tumor regions. Collectively, these results suggested that TFPI2 markedly enhances sorafenib chemosensitivity both *in vitro* and *in vivo*.

### TFPI2 enhances sorafenib sensitivity by activating CCAR2-GADD45A-induced DNA damage

**Figure 7:**
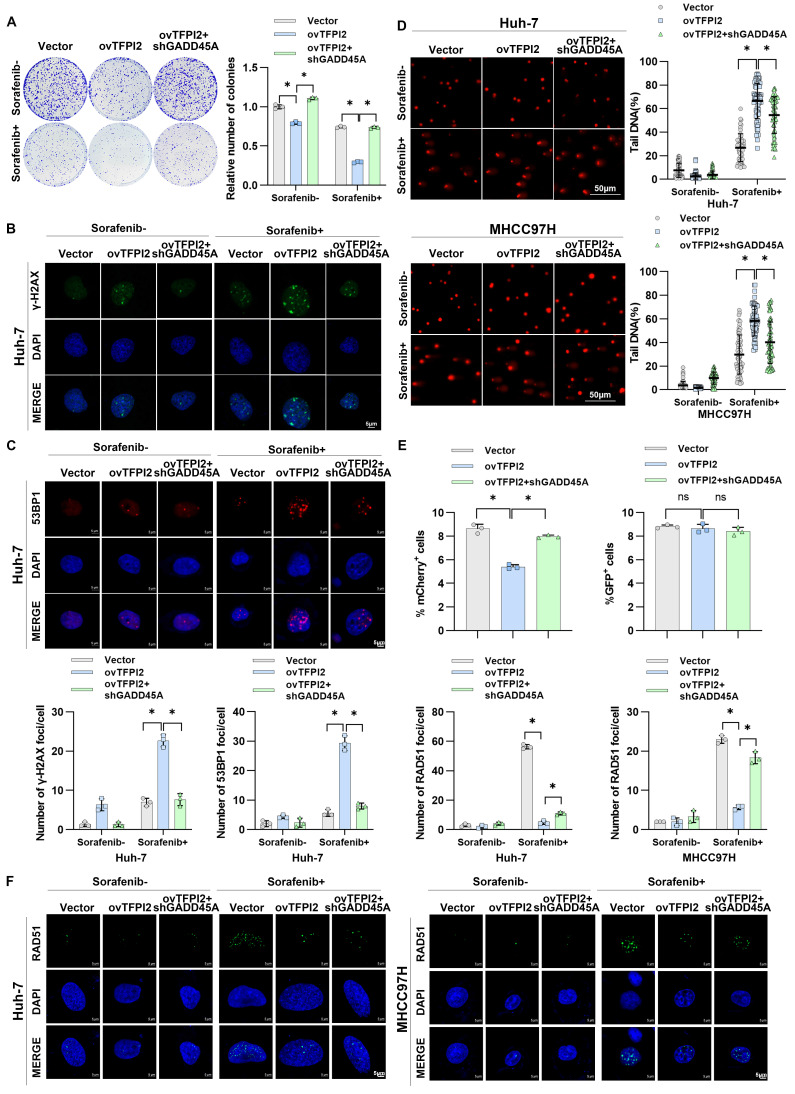
**TFPI2 enhances sorafenib sensitivity via CCAR2-GADD45A-induced DNA damage.** (A) A colony-forming assay assessed the effect of GADD45A knockdown on the colony-forming ability in TFPI2-overexpressing HCC cells with or without sorafenib. Cell colonies were counted using ImageJ. Data are presented as mean ± SEM. *p < 0.05. (B-C) IF staining of γH2AX and 53BP1 foci in GADD45A knockdown, TFPI2-overexpressing cells with or without sorafenib treatment. Scale bar = 5 μm. Foci were counted with ImageJ. Data are presented as mean ± SEM. *p < 0.05. (D) Comet assay images of GADD45A knockdown, TFPI2-overexpressing cells with or without sorafenib. DNA tailing was calculated using ImageJ. Data are presented as mean ± SEM. Scale bar = 50 μm. *p < 0.05. (E) Flow cytometry detected Isel-mediated DNA damage, and NHEJ and HR reporter analyses were performed. *p < 0.05. (F) IF staining and quantitative analysis of RAD51 foci in TFPI2-overexpressing and GADD45A knockdown cells after sorafenib treatment. Count foci with ImageJ. Data are presented as mean ± SEM. *p < 0.05.

We investigated whether CCAR2-GADD45A is involved in TFPI2's influence on chemosensitivity. TFPI2 overexpression increased cell sensitivity to sorafenib, while GADD45A knockdown reversed this effect (**Figure 7A** and **Figure S9A**). GADD45A reportedly plays a pivotal role in DNA repair and is responsible for triggering cell cycle arrest and apoptosis in the presence of DNA damage 31. Therefore, we hypothesized that TFPI2 affects HCC chemosensitivity by regulating GADD45A-mediated DNA damage repair. We observed a higher number of γH2AX and 53BP1 foci in TFPI2-overexpressing cells treated with sorafenib, indicating increased DNA double-strand breaks (**Figure S9B-C**); GADD45A knockdown partly reversed this effect (**Figure 7B-C** and **Figure S9D-E**). The alkaline comet assay showed a significantly higher olive tail moment in TFPI2-overexpressing cells treated with sorafenib, indicating greater DNA damage, which was reversed by GADD45A knockdown (**Figure 7D**). To identify the repair pathway primarily engaged by TFPI2, we employed the HR and NHEJ repair reporter experiment. TFPI2 overexpression significantly inhibited repair effectiveness through the HR pathway rather than through the NHEJ pathway (**Figure 7E**), as evidenced by the decrease in RAD51 foci in TFPI2-overexpressing cells treated with sorafenib (**Figure 7F**). Furthermore, CCAR2 was found to participate in the process through which TFPI2 influences sorafenib sensitivity (**Figure S10A-C**). The above results showed that TFPI2 plays a crucial role in facilitating efficient DNA damage repair through the HR mechanism.

### Polydatin and sorafenib synergistically enhance HCC cell drug sensitivity

**Figure 8:**
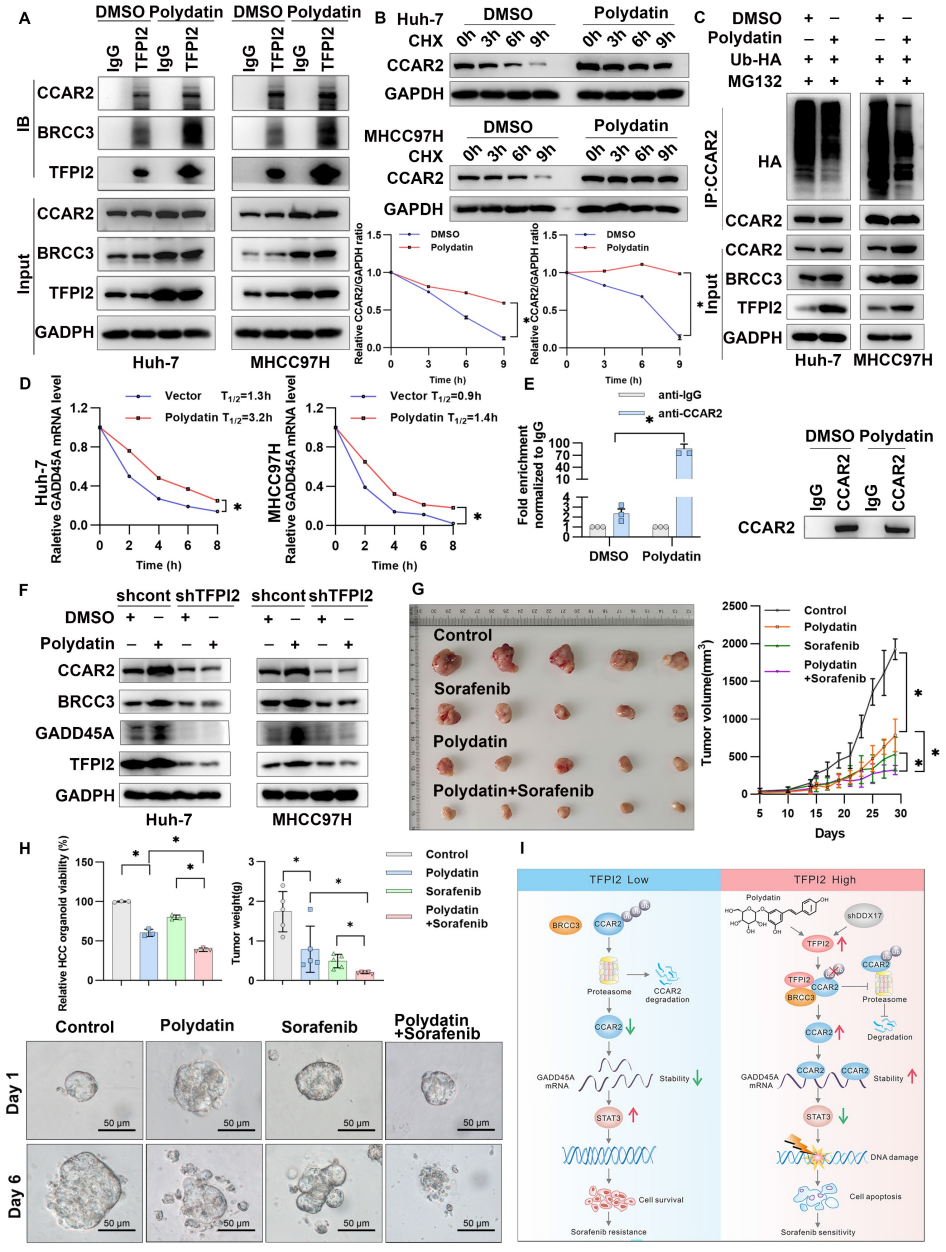
** Polydatin and sorafenib synergistically enhance HCC chemosensitivity.** (A) CO-IP assay to validate the effect of polydatin treatment on TFPI2 binding to BRCC3 and CCAR2. (B) HCC cells were treated with polydatin for 48 hours and then with CHX (300 µM). The cells were harvested at 0, 3, 6, and 9 hours. Western blotting assay to detect the CCAR2 protein stability. Analyze the grayscale values with ImageJ; data are shown as the mean ± SEM. *p < 0.05. (C) HCC cells transfected with HA-tagged ubiquitin (HA-Ub) plasmids (2.5 µg) were treated with polydatin for 48 hours, followed by treatment with 10 µM MG132 for 2 hours. IP assay was used to detect the degradation of CCAR2 protein. (D) RT-qPCR analysis of GADD45A mRNA stability following polydatin treatment (100μM, 48h) and transcriptional inhibition with Actinomycin D (2μg/mL). GADD45A mRNA half-life was calculated at 0, 2, 4, 6, and 8h post-treatment. Data are shown as the mean ± SEM. *p < 0.05. (E) RIP assay examining CCAR2-GADD45A mRNA binding affinity following polydatin treatment (100μM, 48 h). Immunoprecipitation was performed using anti-CCAR2 antibody, with IgG as the negative control. GADD45A mRNA enrichment was quantified by RT-qPCR. Data are shown as the mean ± SEM. *p < 0.05. (F) WB assay to detect the effect of polydatin administration on the downstream signaling axis in TFPI2 knockdown cells. (G) A subcutaneous xenograft mouse model was constructed to establish a polydatin and sorafenib co-administration experiment. Polydatin (100 mg/kg) and sorafenib (30 mg/kg) were administered on alternate days by gavage. Tumor volume was measured over two weeks, with changes shown as a line graph and tumor weight as a histogram. Data are shown as the mean ± SEM. *p < 0.05. (H) HCC organoids verified the effect of polydatin and sorafenib combination on HCC viability, with results shown as a histogram from a 3D cell viability assay. Scale bar: 50 µm. (I) Schematic model: TFPI2 enhances HCC sensitivity to sorafenib by activating the CCAR2-GADD45A-mediated DNA damage repair. DDX17 knockdown upregulates TFPI2 by binding to its promoter. TFPI2 increases GADD45A mRNA stability via CCAR2 and prevents CCAR2 degradation by interacting with BRCC3, promoting DNA repair and apoptosis. Polydatin, targeting TFPI2, synergizes with sorafenib to enhance HCC drug sensitivity.

To further evaluate the potential role of TFPI2 as a therapeutic target for HCC, we conducted a structure-based virtual screen using Schrödinger Maestro 11.4 (**Figure S11A**). The top ten candidates are shown in **Figure S11B,** with representative compounds and their binding modes illustrated in **Figure S11C**. Interestingly, we found that TFPI2 expression gradually increased as the concentration of the polydatin gradient increased; this effect was not observed with other compounds (**Figure S12A**). To explore the effect of polydatin on the BRCC3/CCAR2/GADD45A signaling pathway, we treated cells with polydatin and found that the binding of TFPI2 and BRCC3/CCAR2 protein significantly increased (**Figure 8A**). Additionally, polydatin was found to increase the stability of CCAR2 protein and reduce the ubiquitination level of CCAR2, thus upregulating the expression level of CCAR2 protein (**Figure 8B-C**). Interestingly, polydatin can also upregulate the mRNA stability of GADD45A (**Figure 8D**), and the RIP experiment found that polydatin can enhance the binding of CCAR2 and GADD45A mRNA (**Figure 8E**). To further clarify whether polydatin can specifically target TFPI2, we used molecular docking simulation to show that polydatin specifically binds the amino acid residues LYS-112 and ARG-138 of the KD2 domain of TFPI2 through three hydrogen bonds (binding free energy: -8.5 kcal/mol) (**Figure S12B**). Further, we found that polydatin treatment in TFPI2-expressing cells could upregulate BRCC3 and CCAR2 protein expression, whereas this effect was abrogated in TFPI2-knockdown cells (**Figure 8F**), suggesting that TFPI2 is indispensable for polydatin-mediated activation of the BRCC3-CCAR2 axis. The above experiments suggested that polydatin could activate the BRCC3/CCAR2/GADD45A signaling pathway mediated by TFPI2.

To explore whether polydatin can enhance the sensitivity of sorafenib to HCC cells, we demonstrated that while both sorafenib and polydatin exhibit significant anti-proliferative effects on HCC cells as single agents, their combined effect is more significant, indicating that they synergistically enhance the sensitivity of HCC cells (**Figure S13A-C**). Flow cytometry analysis revealed that polydatin acted synergistically with sorafenib to induce HCC cell apoptosis (**Figure S13D**). To investigate the role of polydatin in chemosensitivity *in vivo*, we subcutaneously injected HCC cells into nude mice. Although both polydatin and sorafenib alone suppressed tumor volume and weight, their combination exerted a more synergistic antitumor effect (**Figure 8G and Figure S13E**). The reduced tumor burden was also validated by Ki67 and PARP immunohistochemistry (**Figure S13F**). Furthermore, combined treatment with polydatin and sorafenib inhibited HCC organoid growth more potently than either treatment alone (**Figure 8H**). These results suggested a promising approach that combines polydatin with sorafenib in treating HCC.

## Discussion

Chemoresistance is a significant barrier to successful HCC treatment 32. Sorafenib, a multikinase inhibitor, is commonly used as a first-line therapy, either alone or in combination with other agents. While initially effective, most patients with HCC develop resistance, making it more challenging to treat 4, 33. Understanding the mechanisms of sorafenib resistance may help identify strategies to mitigate acquired chemoresistance in liver cancer and improve treatment options. Previous studies have shown that cisplatin combined with TFPI2 enhances apoptosis and antitumor activity in NPC cells, and docetaxel with TFPI2 induces greater apoptosis and invasion inhibition than either treatment alone, highlighting its potential in NPC treatment 34, 35. However, TFPI2's role in HCC chemoresistance is unclear. In this study, we found that TFPI2 is downregulated in HCC tissues and cells, and its downregulation may be caused by DDX17-mediated inactivation of the TFPI2 promoter, but the mechanism still needs to be fully elucidated. Furthermore, our results showed that TFPI2 overexpression significantly inhibited HCC cell proliferation and enhanced sorafenib-induced apoptosis, suggesting it is a promising therapeutic target. However, further studies with larger numbers of patients are needed to verify the results in the future.

The GADD45 protein family contributes to diverse cellular functions, including DNA repair, cell growth regulation, and response to genotoxic stress. GADD45A plays a key role in oncogenesis through its anti- and pro-apoptotic activities 31. It acts as a link between p53-dependent DNA repair and cell cycle checkpoints, crucial for maintaining genomic integrity during DNA damage 36. However, p53 promotes GADD45A expression without binding to its promoter, indicating the potential involvement of other processes in protein-protein interactions 37. In our study, GADD45A mRNA levels increased in TFPI2-overexpressing HCC cells, and their knockdown partially reversed TFPI2's inhibitory effect. Persistent DNA damage was observed in sorafenib-treated TFPI2-overexpressing cells, but GADD45A knockdown reversed this damage, indicating that GADD45A-mediated DNA repair affects TFPI2 cell sensitivity to sorafenib. However, the role of TFPI2 in regulating GADD45A expression remains unclear.

Our findings revealed that TFPI2 could enhance GADD45A expression by improving its mRNA stability rather than translation regulation. Since TFPI2 lacks RNA-binding domains, we hypothesized that it participates in RNA regulation by forming a complex with RBPs. CCAR2, an RBP crucial for mRNA stability, possesses the SAP domain, which controls DNA damage response components 38. It can act as a tumor suppressor by promoting p53-mediated apoptosis and reducing mutagenic DNA repair 39. However, CCAR2 may also promote tumor growth by activating oncogenic transcription factors and modulating epigenetic modifiers 38. In this study, TFPI2 overexpression increased CCAR2 binding to GADD45A mRNA, and CCAR2 knockdown reversed the effects of TFPI2 overexpression. TFPI2 also prevents CCAR2 degradation by binding to deubiquitinating enzymes BRCC3 (Figure 8I), suggesting that TFPI2 enhances sorafenib sensitivity through the BRCC3/CCAR2/GADD45A pathway.

Polydatin, derived from the Chinese herb *Polygonum cuspidatum*, has various pharmacological effects 40, 41. Polydatin injection was found to improve lipid peroxidation and DNA damage caused by arsenic exposure while also enhancing the antioxidant defense mechanism 40. Polydatin can suppress the growth of cancer cells in various types of cancer 42, 43. In this study, while our findings establish TFPI2 as the central mediator of polydatin chemosensitization effects in HCC, we acknowledge that the inherent polypharmacology of natural compounds necessitates rigorous off-target profiling. In subsequent studies, we will prioritize implementing systematic off-target evaluations through chemical proteomics with activity-based probes, thermal proteome profiling (TPP), and kinome-wide CETSA integrated with mass spectrometry. Furthermore, the combination of polydatin and sorafenib exerted a synergistic effect in enhancing the sensitivity of HCC cells to sorafenib both *in vitro* and *in vivo*. Importantly, similar results were observed in HCC organoids, suggesting a promising approach for treating patients with HCC by combining polydatin with sorafenib. Nonetheless, the precise mechanisms underlying polydatin-mediated regulation of TFPI2 expression require further investigation for comprehensive elucidation, and the molecular mechanisms underlying the combined treatment of polydatin and sorafenib remain insufficiently explored. Therefore, future studies should further explore the molecular mechanisms of the combined treatment, optimize dosage regimens, and evaluate its long-term safety 44 and efficacy to promote its clinical application in hepatocellular carcinoma therapy.

## Supplementary Material

Supplementary figures.

## Abbreviations

DRR: DSB Repair Reporter
FISH: Fluorescence *in situ* hybridization
H&E: Hematoxylin and eosin
HR: Homologous recombination
IPA: Ingenuity Pathway Analysis
NHEJ: Non-homologous end-joining
OS: Overall survival
PDO: Patient-derived organoids
PFS: Progression-free survival
RBP: RNA-binding proteins
RFS: Relapse-free survival
TFPI: Tissue factor pathway inhibitors
LM: Low magnification
HM: High magnification
LC-MS: Liquid chromatography-mass spectrometry
