# Supplementary material for: Tissue Factor Pathway Inhibitor 2 Enhances Hepatocellular Carcinoma Chemosensitivity by Activating CCAR2-GADD45A-Mediated DNA Damage Repair
Source: Int J Biol Sci. 2025 Jul 11;21(10):4629–46. doi: 10.7150/ijbs.111142 (PMC12320495; doi:10.7150/ijbs.111142)
Supplement: Supplementary file 1 — Supplementary figures. [file ijbsv21p4629s1.pdf]

Supplementary Figure 1

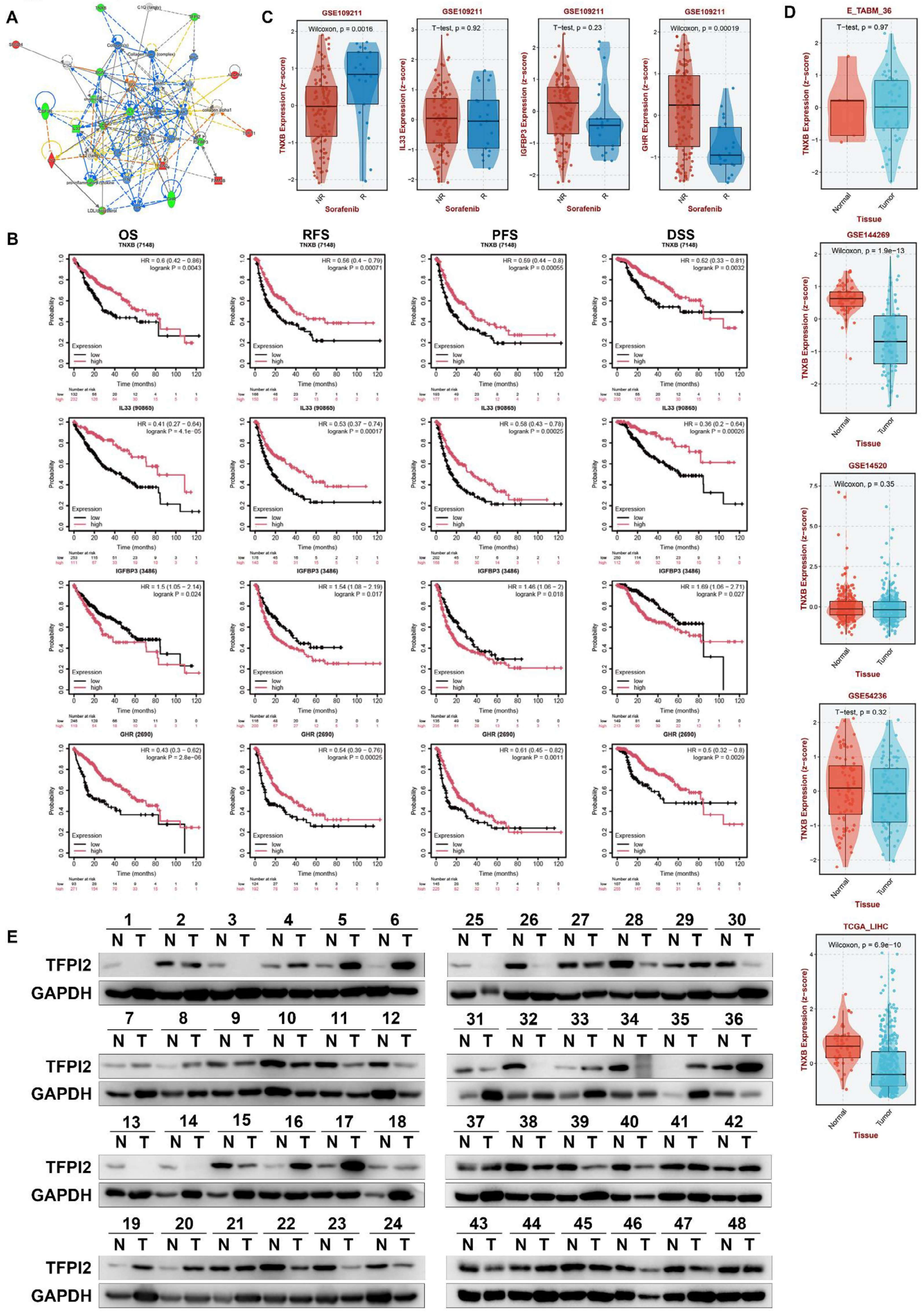

Supplementary Figure 2

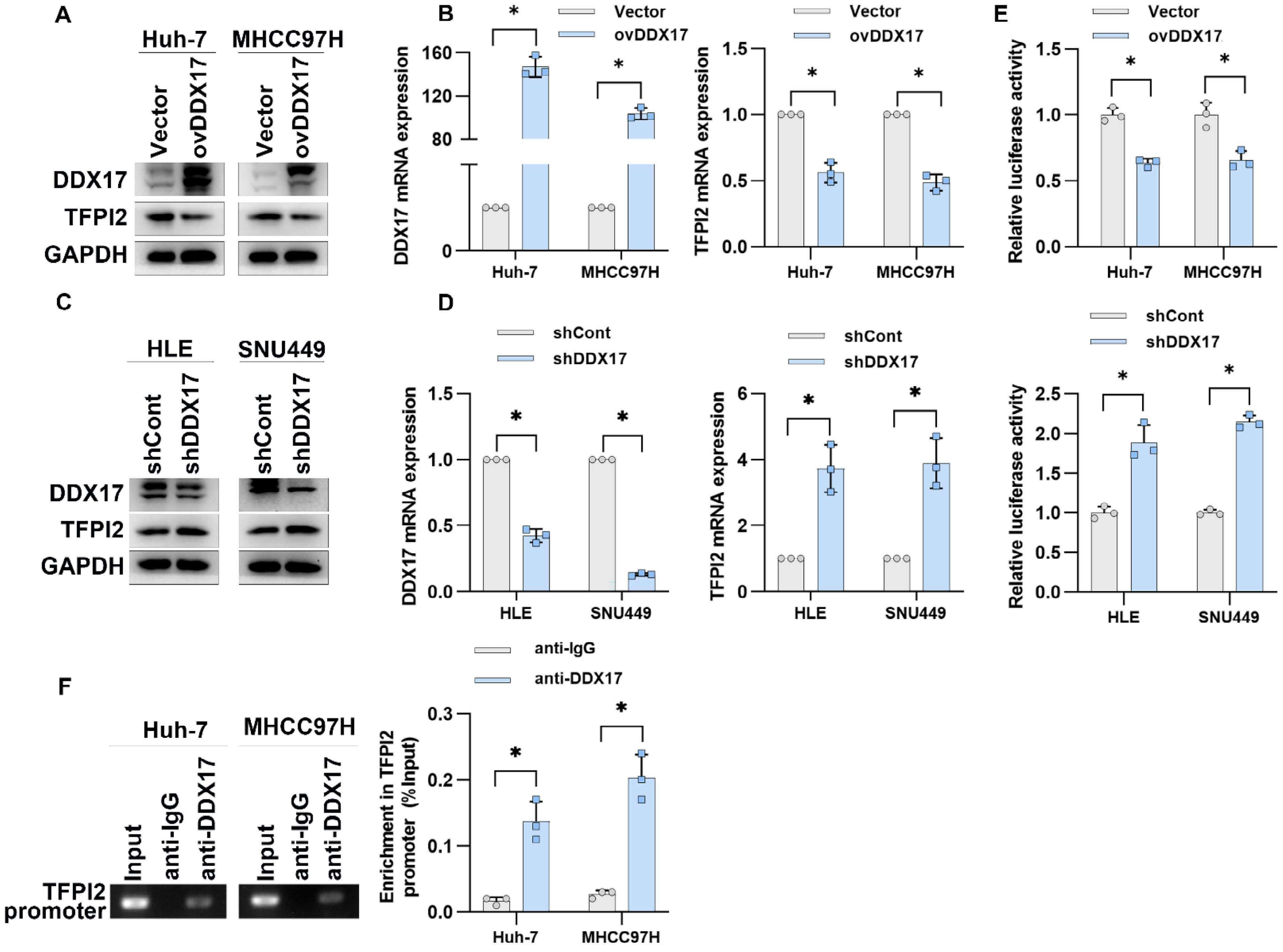

Supplementary Figure 3

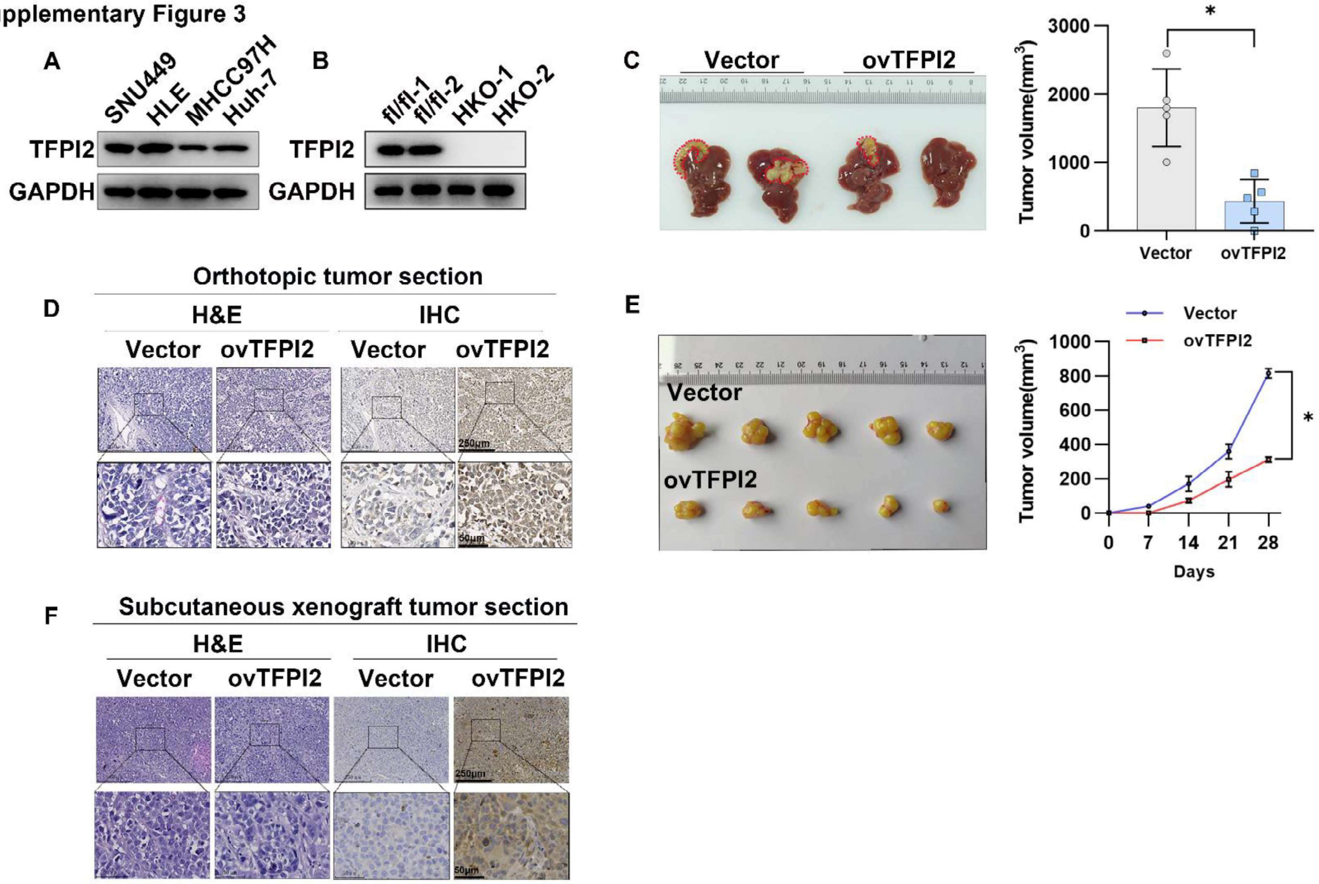

Supplementary Figure 4

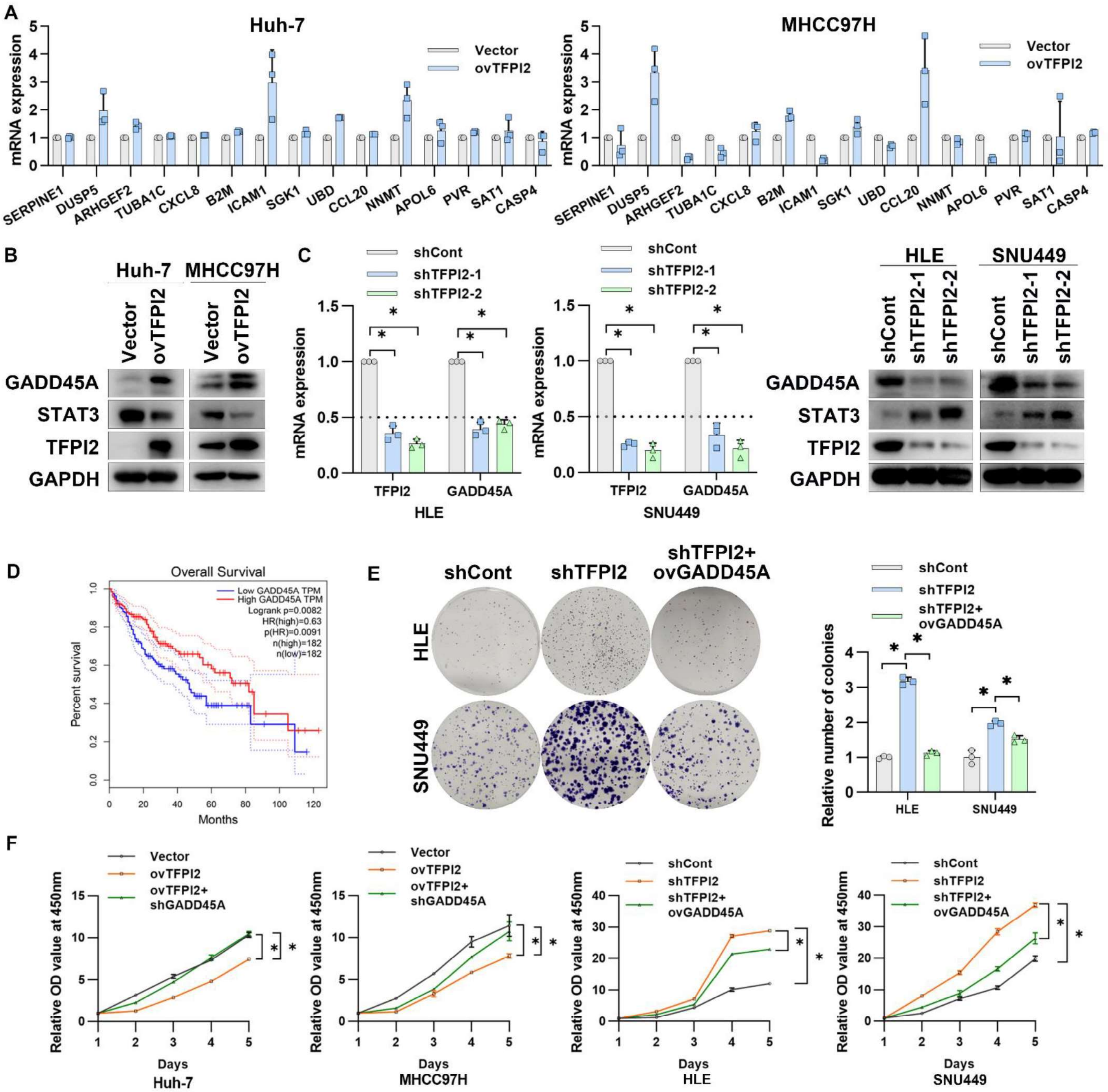

Supplementary Figure 5

A

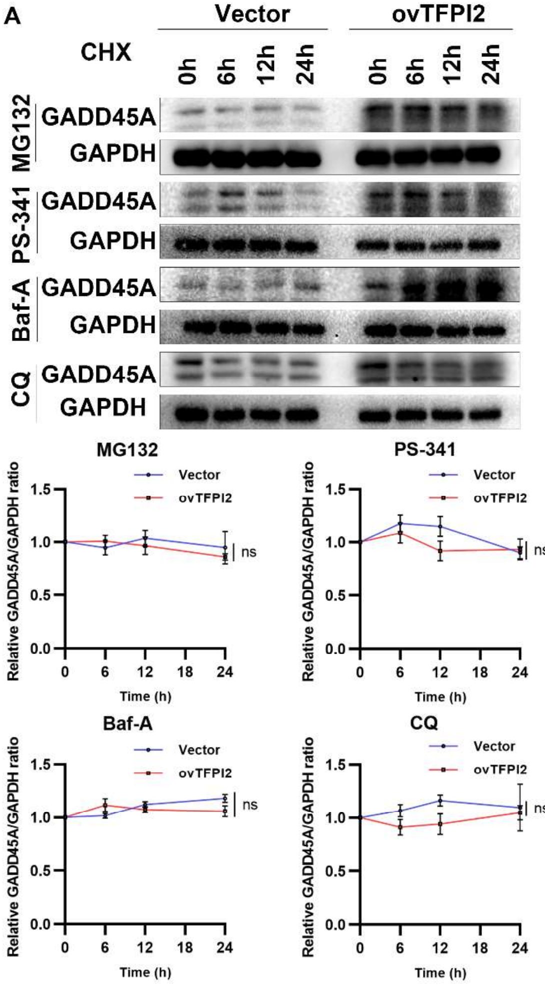

B

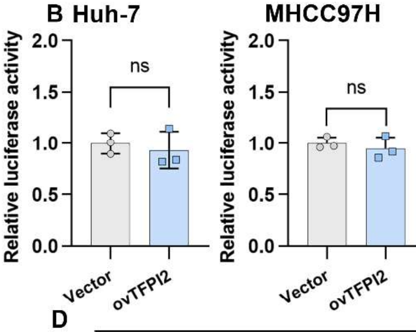

C

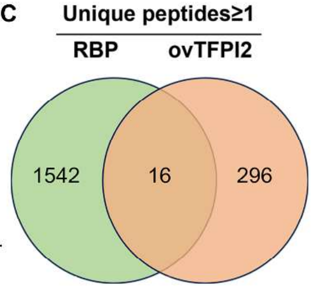

D

GENE	-10lgP	Unique peptides	GENE	-10lgP	Unique peptides
CCAR2	44.45	1	RIOK1	81.16	2
CNOT3	51.99	2	SRRM2	45.42	1
DDX47	63.82	2	ISY1	106.26	3
DHX32	30.1	1	LSM4	58.31	1
ERAL1	39.25	2	NCBP2	40.49	1
GNL1	45	1	PSIP1	38.41	1
GNL3	36.61	1	PUM2	134.11	2
IPO8	73.22	2	IMP4	49.17	1

E

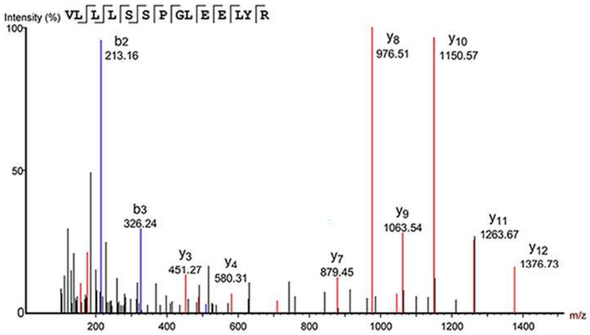

F

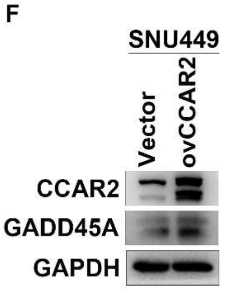

G

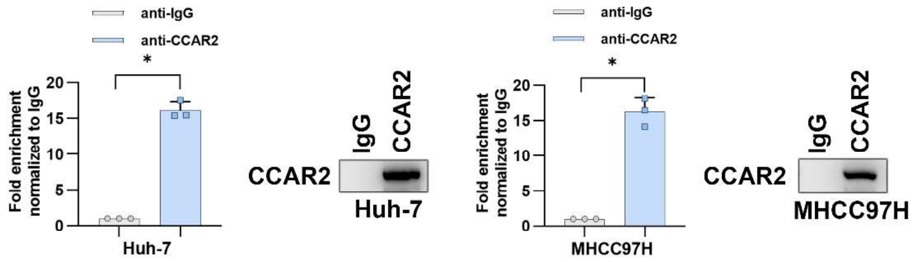

H

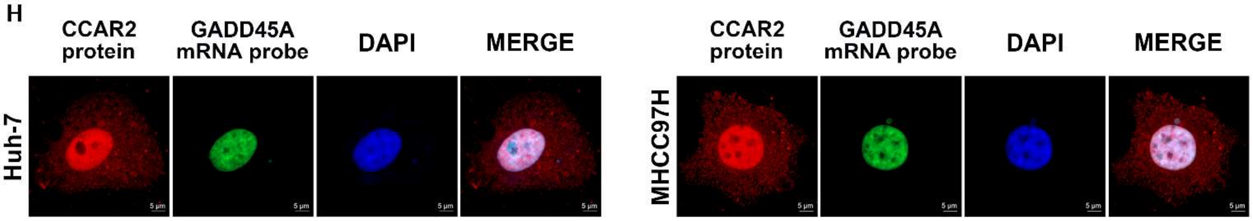

Supplementary Figure 6

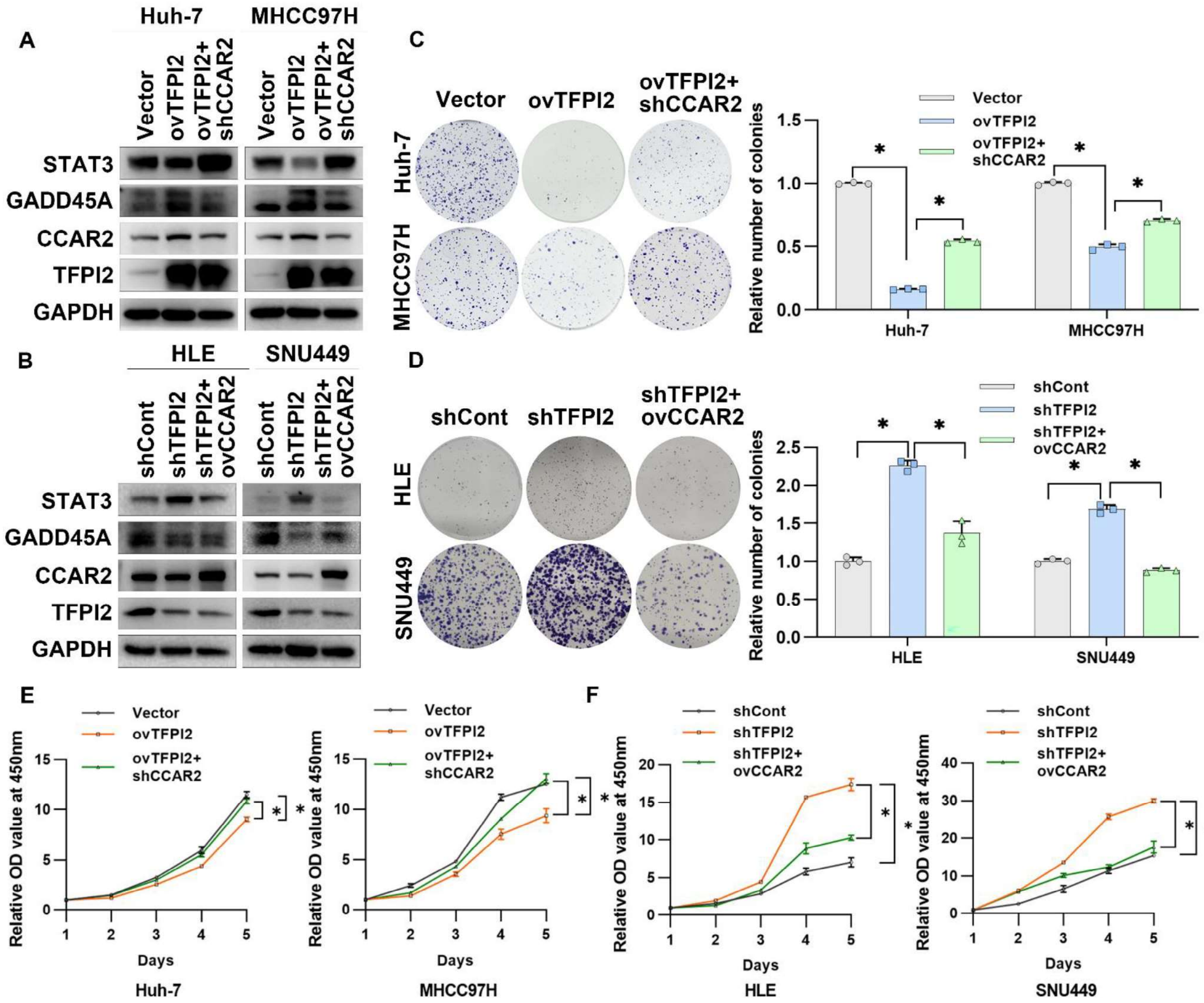

Supplementary Figure 7

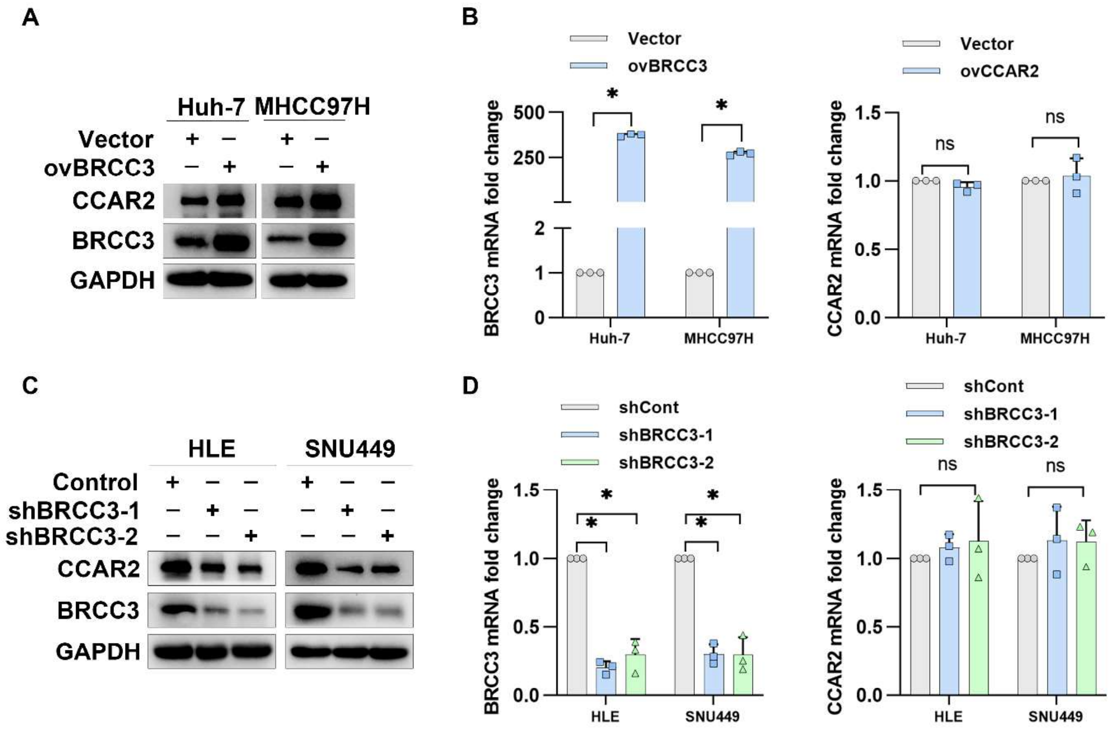

Supplementary Figure 8

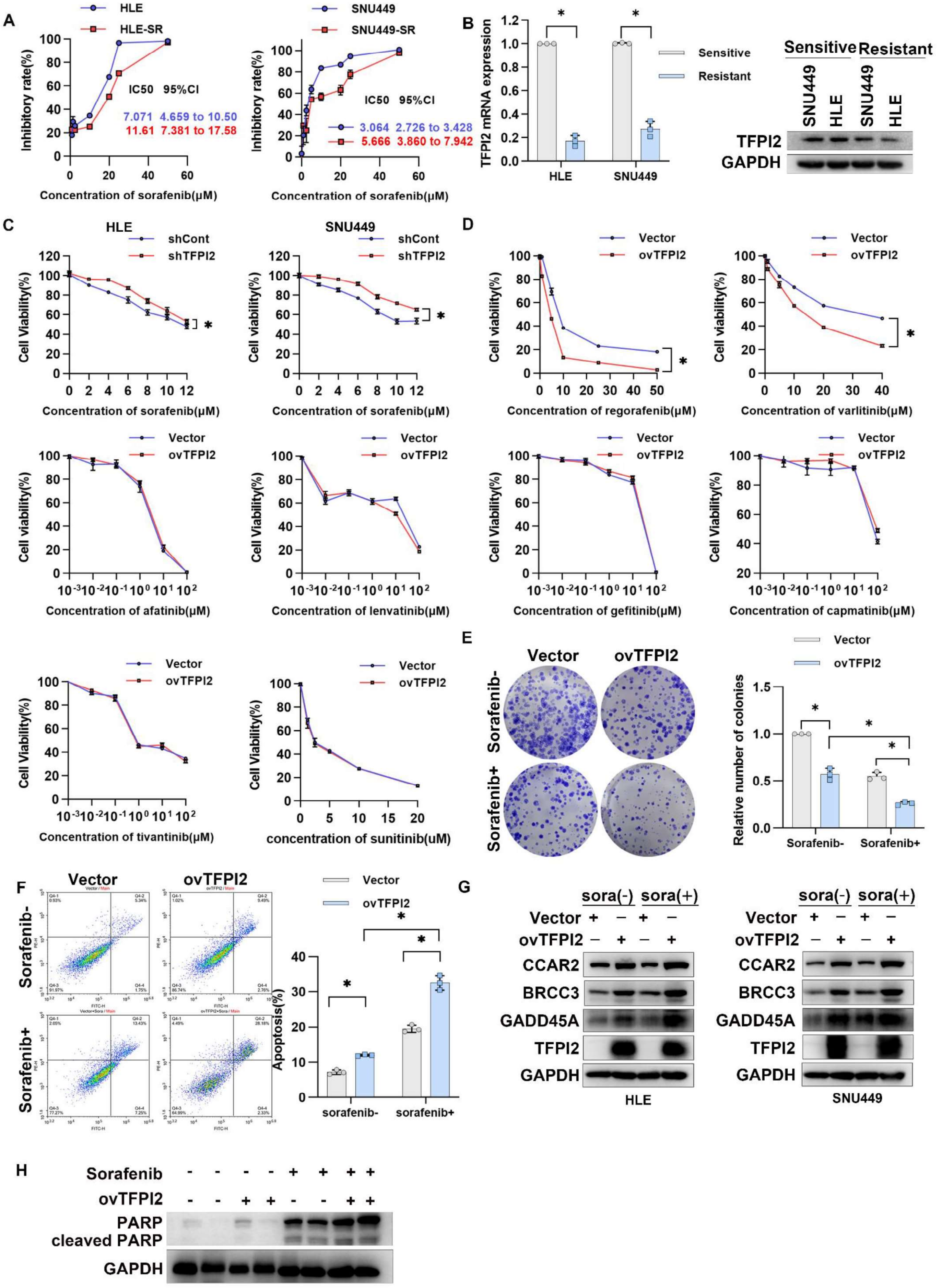

Supplementary Figure 9

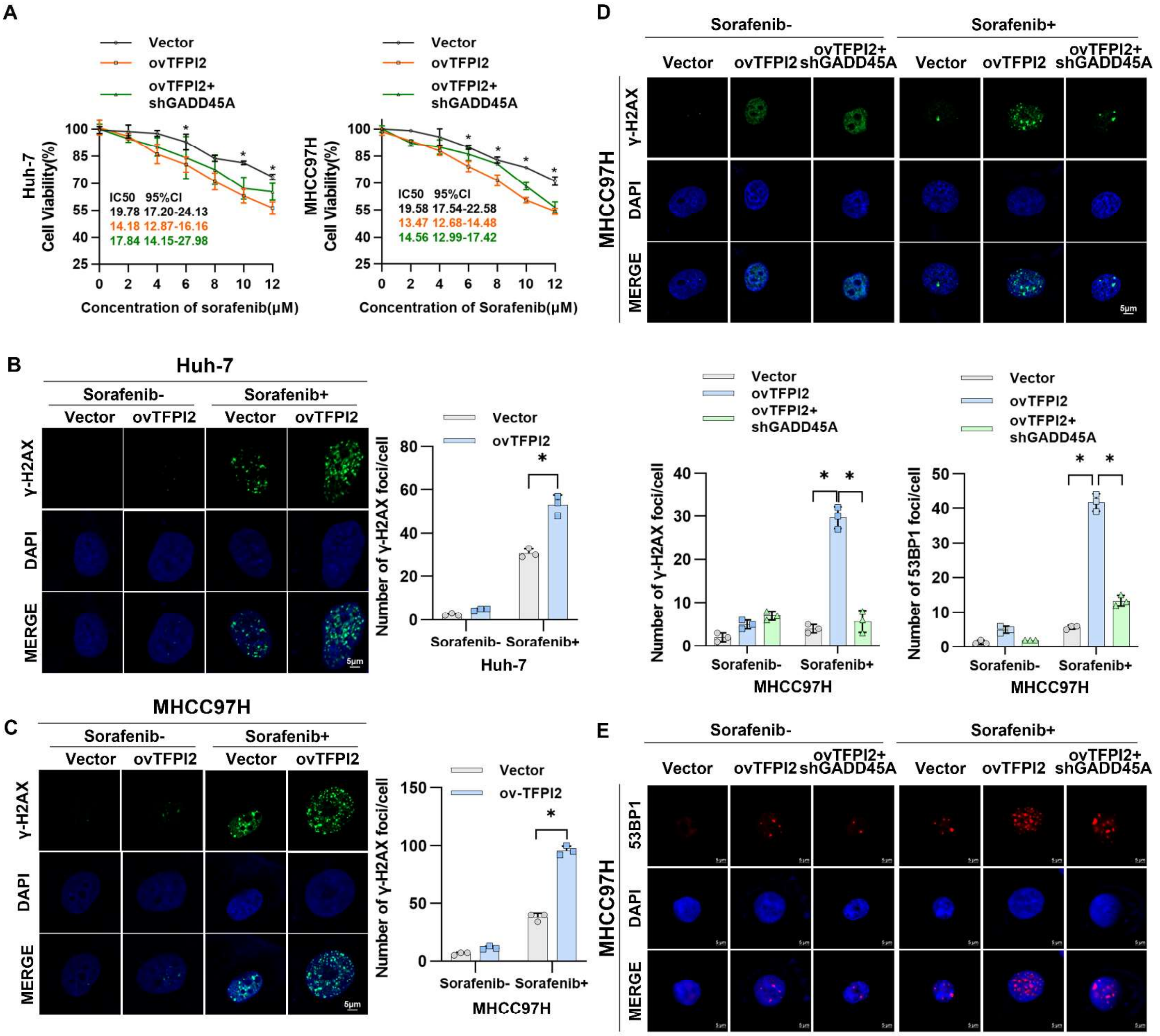

Supplementary Figure 10

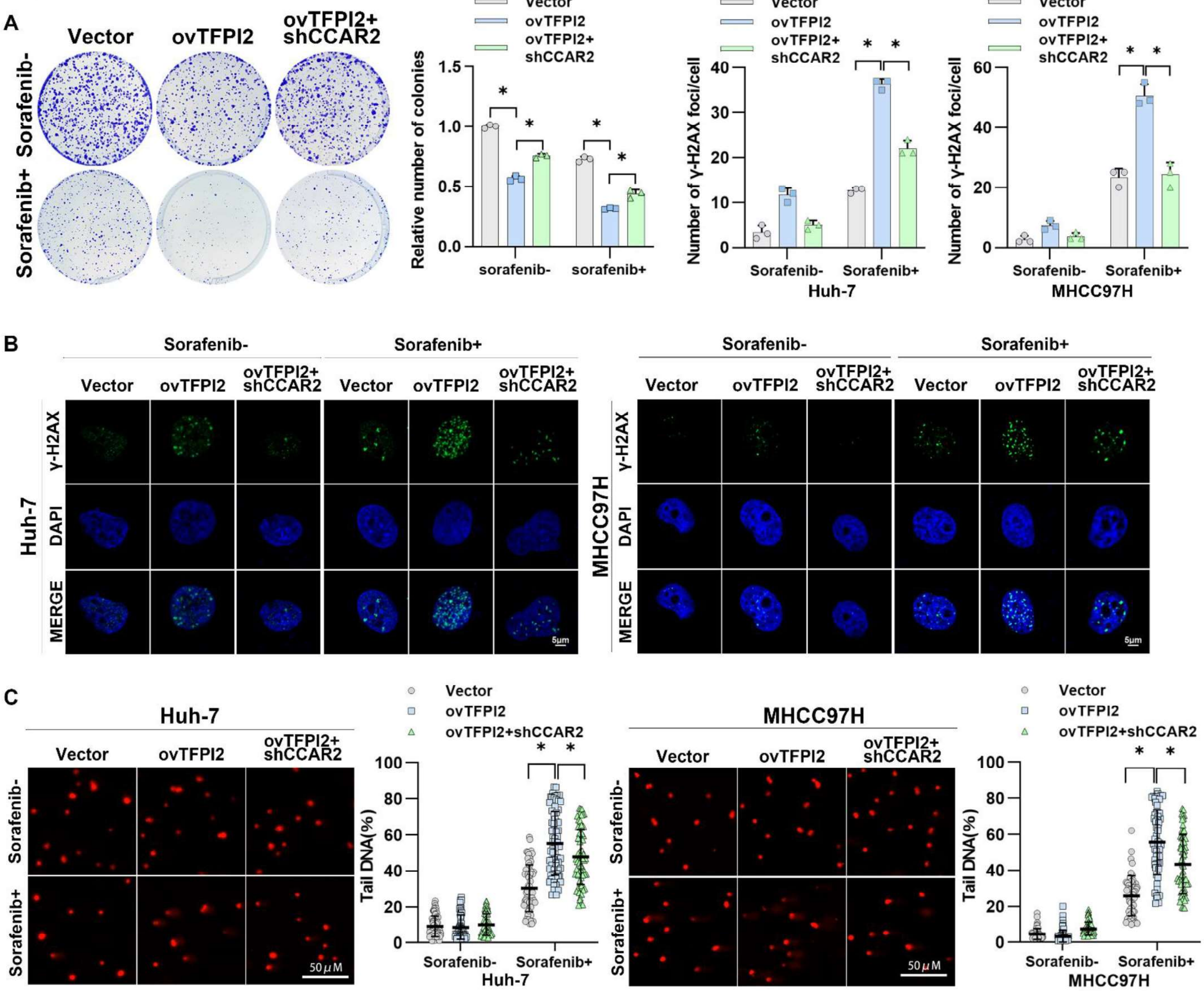

Supplementary Figure 11

A

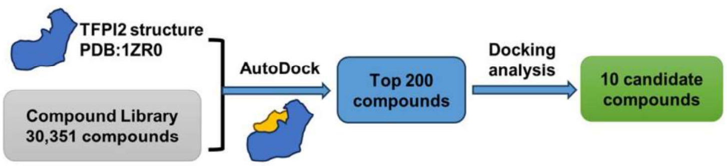

B

Catalog_NO	Name	docking_score	CAS
HY-128851	Coenzyme A	-8.813	85-61-0
HY-N0120	Polydatin	-9.460	65914-17-2
HY-N0272	Eleutheroside E	-9.070	39432-56-9
HY-N5063	Plantainoside D	-11.783	147331-98-4
HY-F0003	NADPH (tetrasodium salt)	-8.384	2646-71-1
HY-N2125	Parishin C	-9.614	174972-80-6
HY-11009	CGP60474	-7.468	164658-13-3
HY-N0071	Crotonoside	-7.666	1818-71-9
HY-14781	Levomefolic acid	-7.485	31690-09-2
HY-13667	Levoleucovorin (Calcium)	-8.220	80433-71-2

C

HY-N0120

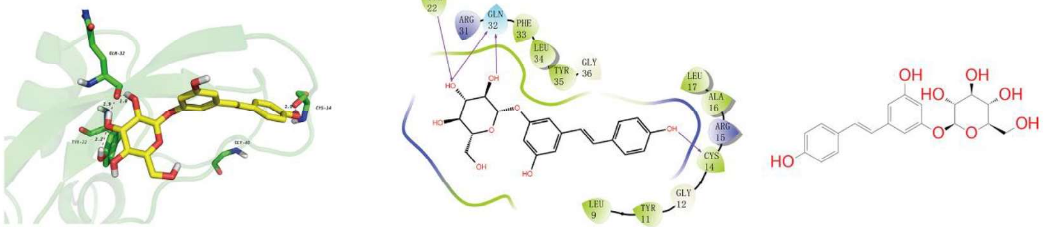

HY-128851

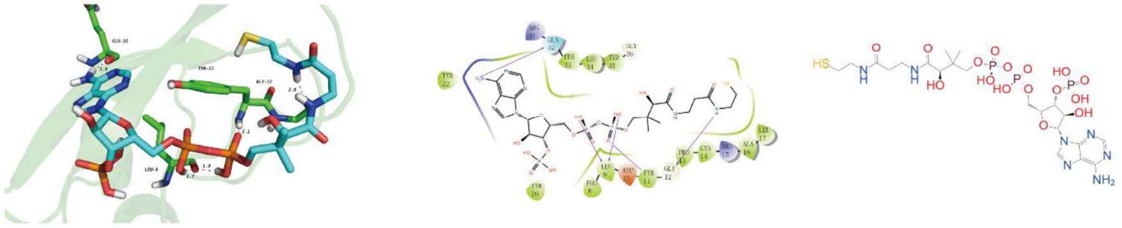

HY-N0272

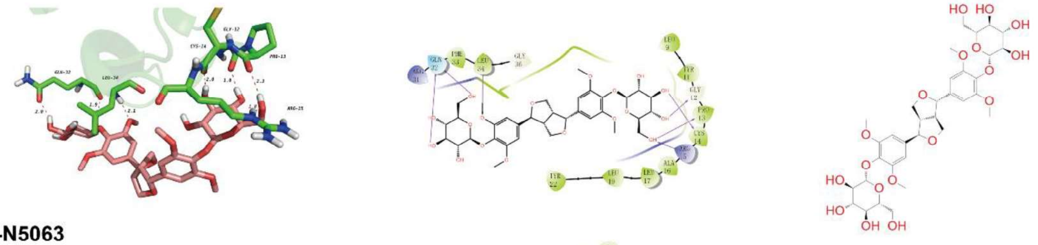

HY-N5063

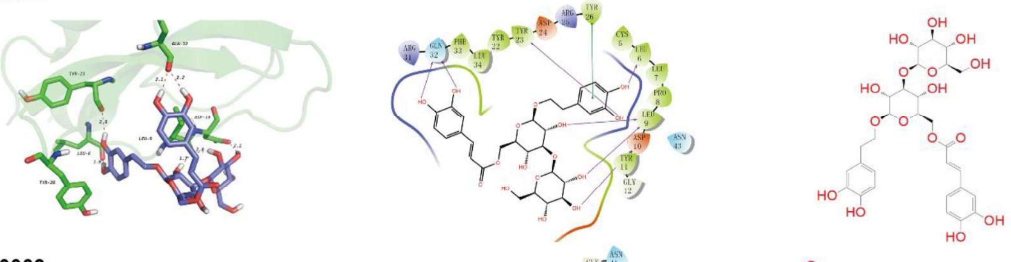

HY-F0003

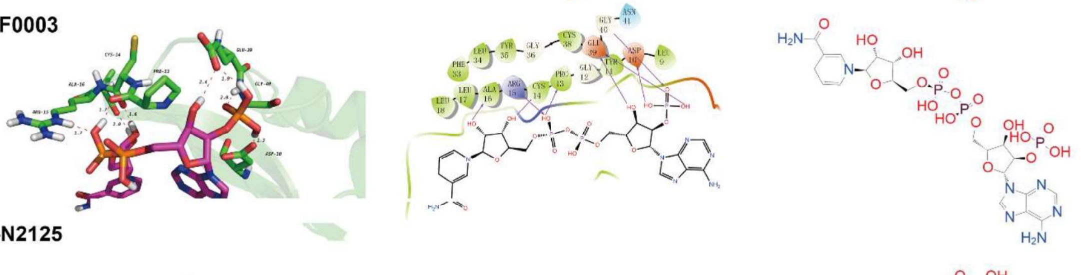

HY-N2125

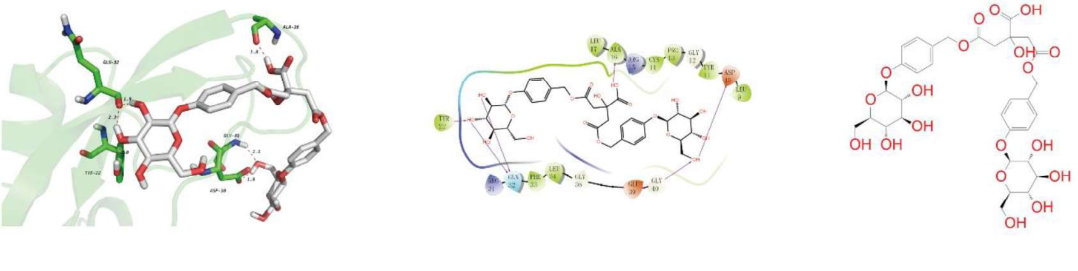

Supplementary Figure 12

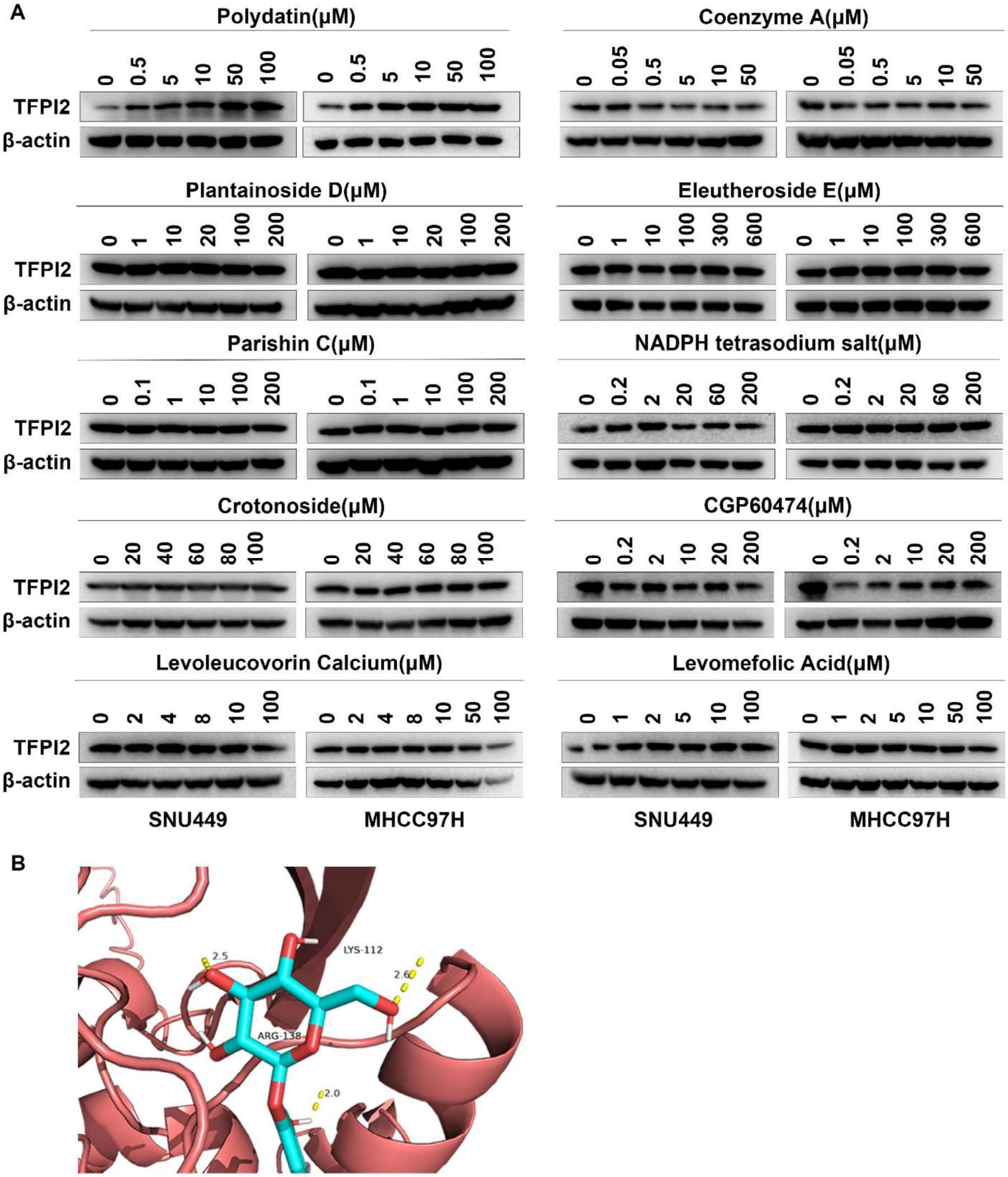

Supplementary Figure 13

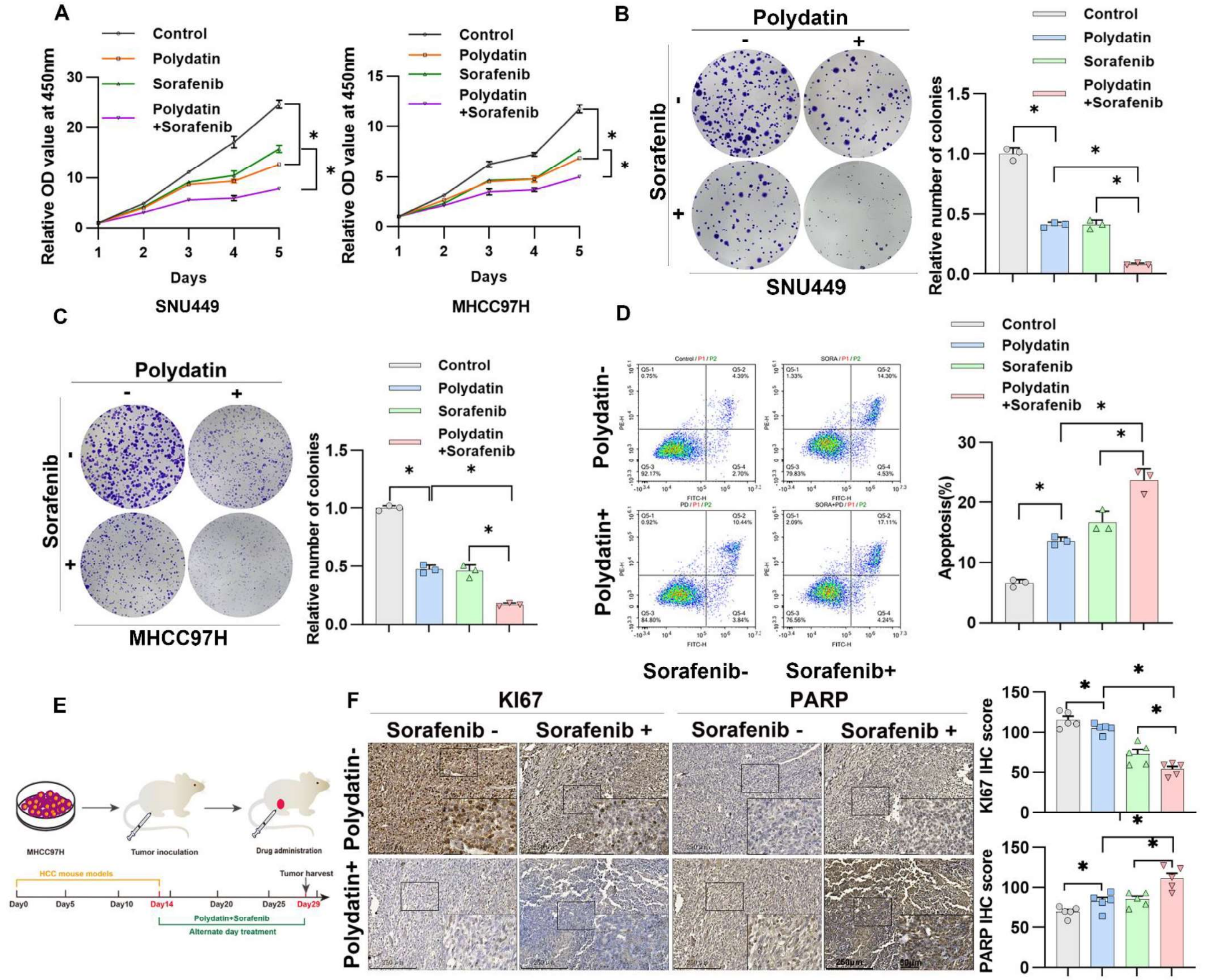

Supplementary Figure 1 TFPI2 was downregulated in HCC. (A) QIAGEN Ingenuity Pathway Analysis (IPA) was used to analyze the potential regulatory genes and pathways of the 38 secreted proteins. (B) Overall survival (OS), relapse-free survival (RFS), progression-free survival (PFS), and disease-specific survival (DSS) curves based on TNXB, IL33, IGFBP3, and GHR expression using the Kaplan-Meier method and analyzed by log-rank test. (C) The GSE109211 dataset was used to analyze TNXB, IL33, IGFBP3, and GHR expression in response to different sorafenib signals. NR: No Response; R: Response. (D) E_TABM_36, GSE144269, GSE14520, GSE54236, and TCGA_LIHC datasets were used to analyze the expression of TNXB in HCC and adjacent tissues. (E) Western blotting assay was used to detect the protein expression of TFPI2 in the tissue samples of paired HCC patients.

Supplementary Figure 2 DDX17 inhibits the transcription of TFPI2 in HCC. (A-D) Western blotting and RT-qPCR were used to detect the effects of overexpression or knockdown of DDX17 on the protein and mRNA expression of TFPI2. Analyze the grayscale values with ImageJ, data were shown as the mean ± SEM. $*p < 0.05$. (E) The dual luciferase reporter system was used to detect the effects of overexpression or knockdown of DDX17 on TFPI2 promoter activity. (F) The ChIP-qPCR technique was employed to identify the TFPI2 promoter DNA region that binds to DDX17.

Supplementary Figure 3 Effect of TFPI2 overexpression in the mouse model. (A)Western blot assay to detect TFPI2 expression in HCC cell lines (SNU449, HLE, MHCC97H, and Huh-7). (B) Western blot analysis of TFPI2 expression in $TFPI2^{fl/fl}$ and $TFPI2^{HKO}$ mice. (C) The orthotopic nude mouse liver cancer model was established by injecting MHCC97H cells infected with control and TFPI2-overexpressing lentivirus into the livers of mice at a concentration of 2×10^6 cells per mouse. Liver tumors

were harvested and measured after 4 weeks. Images of orthotopic xenograft tumors were obtained after the mice were sacrificed. Liver tumor volume was calculated using ruler measurements and statistical analysis. (D) Hematoxylin-Eosin staining and Immunohistochemistry staining of the orthotopic tumor sections. IHC staining was evaluated using histochemical scoring. Data are presented as mean ± SEM. *$p < 0.05$. Scale bars: 250 μm (LM), 50 μm (HM). (E) The subcutaneous xenograft tumor model was established by injecting cells infected with control and TFPI2-overexpressing lentivirus into the subcutaneous region with abundant blood flow in the hind legs of mice. The mice were monitored weekly, and tumor size was measured. After 4 weeks, mice were sacrificed to harvest the tumors, and a tumor growth curve was plotted. (F) Hematoxylin-Eosin staining and Immunohistochemistry staining of the subcutaneous xenograft tumor sections. IHC staining was evaluated using histochemical scoring. Data are presented as mean ± SEM. *$p < 0.05$. Scale bars: 250 μm (LM), 50 μm (HM).

Supplementary Figure 4 The growth inhibition of HCC is regulated by TFPI2 through the modulation of the GADD45A-STAT3 pathway. (A) RT-qPCR was used to validate the differentially expressed genes after TFPI2 overexpression. (B) Western blot was performed to verify the effect of TFPI2 overexpression on the GADD45A-STAT3 pathway. (C) RT-qPCR and Western blot were performed to verify the effect of TFPI2 knockdown on the GADD45A-STAT3 pathway. (D) Kaplan-Meier survival analysis was conducted to assess the impact of GADD45A expression on the overall survival of patients. (E) Clone formation experiments detected the effect on cell colony formation of GADD45A overexpression after TFPI2 knockdown. Count cell colonies with ImageJ, data were shown as the mean ± SEM. *$p < 0.05$. (F) A CCK8 assay was used to detect the effect of GADD45A

knockdown after overexpression of TFPI2 and GADD45A overexpression after TFPI2 knockdown on HCC cell viability.

Supplementary Figure 5 TFPI2 inhibits HCC by increasing the stability of GADD45A mRNA. (A) Western blot analysis of GADD45A protein stability in HCC cells treated with cycloheximide (CHX,300 uM) at indicated time points, in the presence of chloroquine (10 uM), Bafilomycin A1 (500 nM), Bortezomib (PS-341,5 μM), or MG132 (10 μM). The relative expression level of GADD45A was determined based on the intensity ratio of GADD45A/GAPDH. Bands shown for protein stability were quantified using ImageJ. Data were shown as the mean ± SEM. $*p < 0.05$. (B) A dual-fluorescein reporter system to detect the effect of TFPI2 overexpression on GADD45A promoter activity. (C) A Venn diagram was created to screen RNA-binding proteins (RBPs) that bind to TFPI2. (E) The table shows the scores and peptide numbers of the protein. (E) The secondary mass spectrum of peptides was used to identify the CCAR2 protein. (F) Western blot was performed to detect the effect of CCAR2 overexpression on the protein levels of GADD45A. (G) RIP assay to detect the binding efficiency of CCAR2 protein and GADD45 mRNA. (H) Fluorescence in situ hybridization assay to detect the subcellular localization of CCAR2 and GADD45A mRNA. Scale bar = 5 μm.

Supplementary Figure 6 CCAR2 partly reverses TFPI2-mediated HCC growth inhibition. (A) Western blot analysis was conducted to assess protein expression levels of downstream targets following CCAR2 knockdown in TFPI2-overexpressing cells. (B) Western blot analysis was conducted to assess protein expression levels of downstream targets following CCAR2 overexpression in TFPI2-knockdown cells. (C) A colony formation assay was used to detect the effect of CCAR2 knockdown in TFPI2

overexpressing cells on cell proliferation. Count cell colonies with ImageJ, data were shown as the mean ± SEM. *$p < 0.05$.* (D) A colony formation assay was used to detect the effect of CCAR2 overexpression on cell proliferation in TFPI2 knockdown cells. Count cell colonies with ImageJ, data were shown as the mean ± SEM. *$p < 0.05$.* (E) CCK8 assays were applied to evaluate cell viability following CCAR2 knockdown after overexpressing TFPI2 in Huh-7 and MHCC97H cells. (F) CCK8 assays were applied to evaluate cell proliferation following CCAR2 overexpression after TFPI2 knockdown in HLE and SNU449 cells.

Supplementary Figure 7 TFPI2 binds with BRCC3 to affect CCAR2. (A) Western blotting was employed to investigate the impact of BRCC3 overexpression on the protein expression levels of CCAR2. (B) RT-qPCR was employed to investigate the impact of BRCC3 overexpression on the mRNA expression levels of CCAR2. (C) Western blotting was utilized to assess the effects of BRCC3 knockdown on the protein expression levels of CCAR2. (D) RT-qPCR was employed to investigate the impact of BRCC3 knockdown on the mRNA expression levels of CCAR2. Data were shown as the mean ± SEM. *$p < 0.05$.*

Supplementary Figure 8 TFPI2 enhanced the sorafenib sensitivity of HCC *in vitro*. (A) CCK8 was used to detect the inhibition rate of sorafenib in Sorafenib-resistant and control cells. The parental HCC was used as a control. (B) RT-qPCR and western blot analyses were conducted to detect the expression of TFPI2 mRNA and protein in sorafenib-resistant and sorafenib-sensitive HCC cells. (C) CCK8 assay was used to detect the sensitivity of TFPI2 knockdown to sorafenib in HCC cells. (D) The CCK8 assay was used to determine the sensitivity of TFPI2 overexpression to regorafenib, varlitinib, afatinib

dimaleate, lenvatinib, gefitinib, capmatinib, tivantinib, and sunitinib in HCC cells. (E) Colony formation assay was used to verify the effect of TFPI2 overexpression on cell proliferation under sorafenib treatment. Count cell colonies with ImageJ, data were shown as the mean ± SEM. *$p < 0.05$. (F) Flow cytometry was used to detect the role of TFPI2 overexpress in the process of sorafenib-induced apoptosis. (G) Western blotting was utilized to assess the effects of sorafenib treatment on the protein expression levels of TFPI2 downstream signal axis. (H) Western blotting was utilized to assess the effects of sorafenib treatment on the protein expression levels of PARP and cleaved PARP expression levels *in vivo*.

Supplementary Figure 9 TFPI2 enhances the drug sensitivity of HCC cells to sorafenib through GADD45A-induced DNA damage. (A) A CCK8 assay was performed to explore the effect of GADD45A knockdown after TFPI2 overexpression for 48h on cell viability after treatment with different sorafenib concentrations. (B-C) Immunofluorescence staining of γH2AX foci in TFPI2 overexpression HCC cells with or without sorafenib treatment. Count foci with ImageJ. Data are presented as mean ± SEM. Scale bar = 5 μm. *$p < 0.05$.(DD) Immunofluorescent staining of γH2AX foci in GADD45A knockdown TFPI2-overexpressing cells with or without sorafenib treatment. Count foci with ImageJ. Data are presented as mean ± SEM. Scale bar = 5 μm. *$p < 0.05$. (E) Immunofluorescent staining of 53BP1 foci in GADD45A knockdown TFPI2-overexpressing cells with or without sorafenib treatment. Count foci with ImageJ. Data are presented as mean ± SEM. Scale bar = 5 μm. *$p < 0.05$.

Supplementary Figure 10 CCAR2 is involved in the process of TFPI2 enhancing the drug sensitivity of HCC cells to sorafenib through GADD45A-induced DNA damage (A) Clone formation experiments detected the effect on cell colony formation of CCAR2 knockdown after TFPI2

overexpressing with or without sorafenib treatment. Count cell colonies with ImageJ, data were shown as the mean ± SEM. **p* < *0.05.* (B) Immunofluorescent staining of γH2AX foci in CCAR2 knockdown TFPI2-overexpressing cells with or without sorafenib treatment. Scale bar = 5 μm. (C) Representative images of the comet assay of CCAR2 knockdown in TFPI2-overexpressing cells with or without sorafenib treatment. DNA tailing was calculated using ImageJ. Data are presented as mean ± SEM. Scale bar = 50 μm. **p* < 0.05.

Supplementary Figure 11 Screening TFPI2 activated drugs (A) Schematic overview: The three libraries compounds containing （MCE Bioactive Compound Library Plus,19617 compounds, HY-L001P)、（Anti-Cancer Compound Library, 6839 compounds, HY-L025)、（Metabolism/Protease Compound Library，3895 compounds, HY-L012）were used for virtual screening with the structure of TFPI2. (B) Docking scores for the top 10 candidate compounds. (C) Polydatin binds to the active pocket of TFPI2 through six H-bonds.

Supplementary Figure 12 The pharmaceutical polydatin has a synergistic effect with sorafenib on HCC cells. (A) Western blotting was used to detect the effect of different concentrations of drug treatment on TFPI2 expression levels. (B) Molecular docking of polydatin with TFPI2 protein was performed using Pymol software, showing binding sites and interaction patterns. Molecular docking results demonstrated that polydatin exhibited the highest binding affinity with TFPI2 (ΔG = -8.2 kcal/mol) among all components in the regulatory axis.

Supplementary Figure 13 Polydatin acts specifically on TFPI2 and is synergistic with sorafenib in the treatment of HCC. (A) A CCK8 experiment was performed to monitor changes in the viability of HCC cells within 5 days after using Polydatin or sorafenib alone and a combination of both. (B-C) A colony formation assay was used to verify the effect of polydatin or sorafenib alone and their combination on the colony formation of HCC cells. Count cell colonies with ImageJ, data were shown as the mean ± SEM. *$p < 0.05$.* (D) Flow cytometry was used to detect the effect of polydatin or sorafenib alone and in combination on apoptosis. (E) Schematic diagram illustrating the dosing regimen administered to the subcutaneous xenograft mouse model. Nude mice administered MHCC97H cells subcutaneously to establish the xenograft tumor model were divided into 4 groups after 4 weeks of the injection: control, polydatin, sorafenib, and polydatin combined with sorafenib administration groups. Polydatin (100 mg/kg) and sorafenib (30 mg/kg) were administered on alternate days by gavage, and tumor volume was measured. (F) Representative images of Ki67- and PARP-stained sections for immunohistochemistry assessment, with the staining intensity scored and counted in tumor tissue sections of mice. IHC staining was evaluated using histochemical scoring. Data are presented as mean ± SEM. *$p < 0.05$. Scale bars: 250 μm (LM), 50 μm (HM).

Supplementary Table 1: Correlation between TFPI2 expression and clinicopathological characteristics

characteristics	Total(n=60)	TFPI2 protein expression		*p-value*
		High	Low	
Gender	60			1.0
Male	48	9	39	
Female	12	2	10	
Age, years				0.744
⩾50	37	7	30	
<50	23	3	20	
ALT/AST				0.168
⩾1	45	10	35	
<1	15	5	10	
AFP (ng/ml)				**0.043***
⩾400	49	7	42	
<400	11	5	6	
Tumor Size (cm)				**0.030***
⩾3	17	5	12	
<3	43	6	37	
Tumor Stage				0.428
N1	49	11	38	
N0	11	3	8	
Tumor invasiveness				0.183
0	50	7	43	
1	10	3	7	

$p<0.05$ indicates a signifcant association among the variables

Supplementary Table 2: The primer used in the paper

Gene	Primer
TFPI2-F	GCTCAGGAGCCAACAGACTA
TFPI2-R	CAAGCCTCCCAGGTGTAGAA
GAPDH-F	TATGACAACAGCCTCAAGAT
GAPDH-R	AGTCCTTCCACGATACCA
BRCC3-F	ATAAGAGGAAGGACCGAGTA
BRCC3-R	GATGTGTAAGGCTGTGGAT
CCAR2-F	CAAGGAAGAAGCCACCAA
CCAR2-R	CAAGTAGCCACACCAGTT
CNOT3-F	AGAAGGAGAAGGAAGAGGTT
CNOT3-R	CTGCTGGACTGGTTGAAG
DDX47-F	GAGTGCTGTGATTGTAGGT
DDX47-R	GTATTCGGTCGGCTTCAT
DHX32-F	TGGTGTCGTGATTACTTCC
DHX32-R	ACTTGCTGTAGAATGTCCTT
ERAL1-F	AACAGAGATGAGCAGGATG
ERAL1-R	CACAAGAACCACAACAAGAT
GNL1-F	CATCATATCCGCAGGCTTA
GNL1-R	CCAAGTTCTCCAGTCACATA
GNL3-F	CAGAGCATAAGAGCCATCA
GNL3-R	AGACCTTGTAGACTGTTCAC
IP08-F	TTGGTCCTACTGCCTACTT
IP08-R	CGTGCGGTATTGGTGTAA
RIOK1-F	GTCCAGTTATCAGAATCCAAG
RIOK1-R	AACACCTCTTCATCCACAT
SRRM2-F	ACACCAAGACGAGGAAGA
SRRM2-R	TTAGGATGAGGAGATGAACTG
ISY1-F	AGAAGTGGAGACGACAGAT
ISY1-R	GGCACAATAACACCATCATC
LSM4-F	GAACATTAACCTGCGAGAAG
LSM4-R	CACCTCCTCCTTGACCAT
NCBP2-F	GGTGACAATGAAGAACAAGA
NCBP2-R	TTCCATAGCCTCCTCTCC
PSIP1-F	GAGGCAGGAGTAGTGACA
PSIP1-R	CAGAATCGGAGGTTGAAGTA
PUM2-F	TGGAATGGATGCCGATTG
PUM2-R	GCTGTAGGATTAGGAAGAGG
PSMD1-F	AAGAACGCCAGCAATGAT
PSMD1-R	TACCAGAGCCACAATAAGC
GOLIM4-F	ACATTCTCCACAAGGTTACA
GOLIM4-R	TTCTCTGCGTTCCACTTC
CHD2-F	CTGGAGAAGGAGTTGATGAA

CHD2-R	TGTGCTGTGGATGTAAGAC
ARHGAP35-F	AGTGTGACGAAGGTGTTG
ARHGAP35-R	CTCAGCCAGTTCTCATTGT
KRT8 -F	GGAGCAGCAGAACAAGAT
KRT8 -R	GGTTGGCAATATCCTCGTA
LUC7L3-F	AACTGCTAAGGTCCACAAC
LUC7L3-R	GTCTGATGTTCGGCTTGA
MANF-F	TCGGTTGTGCTACTATATCG
MANF-R	CATATCTGGCTGTCCTTCTT
SRRM1-F	GCTCATCATCCTCTTCATCT
SRRM1-R	CCAACCTCACCATTCTTCA
SRRT-F	AGCAGGAAGTGGAGAAGT
SRRT-R	AGCATCAGTGAGGAAGTTG
LMNB2-F	GAACAACTCGGACAAGGAT
LMNB2-R	TCTCATTCTCACGCATCAC
MCM7-F	GTGGAGAACTGACCTTAGAG
MCM7 R	CGTATGCTGCTGTGATGT
ALDH3A2-F	TGCTTGAGAATCTTCCTGAA
ALDH3A2-R	TGGCTGAATGGTGAGAAC
RSRC2-F	AGACACAGGCATAGGACTA
RSRC2-R	AGCAACATTGAGAACAGAAC
ARID4A-F	AAGTTGTAAGTGTGGTGTCT
ARID4A-R	CTGCTGGAGGAAGTTGTC
MAP3K9-F	ATGGAAGACTGCTGGAATC
MAP3K9-R	GCGGCTGTTGATAAGACT
TRIM28-F	CCGTCTTCAAGGTCTTCC
TRIM28-R	GAGCCATAAGCACAGGTT
TPX2-F	TACAGGAGGAAGAGCAGAA
TPX2-R	GATACAGGCACAGTCAGAG
UBN1-F	CCAGTAAGGAGAAGAAGAAGA
UBN1-R	AGTCGTTGTCAGAAGTAGAG
TACC3-F	TCCTTCTCCGACCTCTTC
TACC3 R	TCTGCTCCACTGTCTTCT
HMGCS1-F	TTGATAGAGATGTGGAGAAGG
HMGCS1-R	GCTAATTGCTGAGGTGAGT
EIF5-F	GACCAGTTCTATCGCTACA
EIF5-R	GATTCTCACATTCAGGACAG
SND1-F	GAAGCAGATGGCAGTGAA
SND1-R	GGTGAATCGTCTTGTAATCG
SPAG5-F	AAGGCAGCAACAACTCAT
SPAG5-R	CTCAGATACAGCAGCAACT
TBX3-F	GCCTACCATCCGTTCCTA
TBX3-R	CGTCCTCCACCTCTTCTT

GATA4-F	TCAGTCAGTGCGATGTCT
GATA4-R	CAGGCTGTTCCAAGAGTC
PEG10-F	CTGAGGATGAGGATGATGAC
PEG10-R	CTGAATGTGGTCGCTGAG
ALKBH5 F	GTTCCAGTTCAAGCCTATTC
ALKBH5 R	CGCCAGTAGTTCTCAGAG
TCAF1-F	CCATTCATCCGTCTCTTCA
TCAF1 R	GGTAGCCACTTCCTTCTG
HSP90B1-F	CAAGGACGGATGATGAAGT
HSP90B1-R	CAAGGAAGGCGGAATAGAA
RPL36A−HNRNPH2-F	GTTATGACAGGAAGCAGAGT
RPL36A−HNRNPH2-R	AACGCACTCAAGCCTTAG
TFPI2-F	CCTACTTCTCCGTTACTACTAC
TFPI2-R	GTCGTCCACACTCACTTG
TYW1B-F	GCATCACAGAGACACCAA
TYW1B R	AGAGCAGGAGTTATCATAGC
CREBRF-F	AGGAGGATGTTGATGATGAG
CREBRF-R	TGCTGTGATGGTGTAAGTT
HYOU1-F	GATGAGATTGAGCAGGTGAT
HYOU1-R	CGTAGTTGATGTGGAAGTTG
PDIA4-F	CCAGCCTACCAGCAATAC
PDIA4-R	AGCACGAAGTCCTTGATG
FRS2-F	ACATACAGGTTGACTTGGAA
FRS2-R	GACATAGCAGCAGTTCTCT
HSPA5-F	GGAGGAGGACAAGAAGGA
HSPA5-R	AGTGAAGGCGACATAGGA
GADD45B-F	AGGAGGAGGAGGATGACA
GADD45B R	CGTGACCAGGAGACAATG
SH3BP5-F	GAGATGCCTGACCAGTTC
SH3BP5-R	CTCCTCGTTCTACTTCACAT
CRELD2-F	GAAGAGCGAATATCCTGACT
CRELD2-R	GCCAACATCCAAGAGACA
GADD45A-F	GAGGACGACGACAGAGAT
GADD45A-R	ATTGATCCATGTAGCGACTT
SDF2L1-F	ACCTCTGTGTTCCTGTCA
SDF2L1-R	CACTAGGCTTGATGAAGATG
SEC24A-F	CTACTACACCAATGCCTTCT
SEC24A-R	AGCCACCTCCTTCAATCT
ARID4B-F	GATGGAGGAGGAGAGGAAT
ARID4B-R	CATACCGCACTTGGACTT
HERPUD1-F	AATGCTGCTCCTCAAGTG
HERPUD1-R	GCTGCTGAATAGGTCCAAT
KLF10-F	CAGTGGCAGATGTTGATGA

KLF10-R	GATGTGACTCCTTATCCTTGA
WHAMM-F	CTCTGTCCTCATCCTCTCA
WHAMM R	GTCCTGCTCATCACTGTC
NUPR1-F	CTGGATGAATCTGACCTCTAT
NUPR1-R	CCACCTCCTGTAACCAAG
TGM2-F	CCACATCACCAACAACAC
TGM2-R	GTCCATTCTCACCTTAACTTC
NNMT-F	ACTACTCAGACCAGAACCT
NNMT-R	TTCACAGCAGCCTCTACT
SPINK1-F	GTCTATCTGGTAACACTGGAG
SPINK1-R	ACATAACACGCATTCATTGG
UBE2L6-F	GACGAGAACGGACAGATT
UBE2L6-R	GTCTATTCACCAGCACATTG
THBS1-F	GCTCAGTATGACTATGACAGA
THBS1-R	CAGGAATGCCATCGTTGT
AREG-F	GTGCTGTCGCTCTTGATA
AREG-R	TGCTTCCAGGTGCTCTAT
LDLR-F	CAGCGAAGATGCGAAGAT
LDLR-R	AGAAGAGGTAGGCGATGG
HAX1-F	AGCATCTTCAGCGATATGG
HAX1-R	CTGGTGACTATCTGGATACTTA
CXCL5-F	CCACGCAAGGAGTTCATC
CXCL5-R	GGCTTCTGGATCAAGACAA
ICAM1-F	CGAGGTGACAGTGAAGTG
ICAM1-R	GTCTGGTTCTTGTGTATAAGC
DUSP6-F	AAGTTCCAAGCCGAGTTC
DUSP6-R	TACCAAGACACCACAGTTC
SGK1-F	GAACACAACAGCACAACAT
SGK1-R	CCATACAGCATCTCATACAAG
UBD -F	AGAGAAGACCATCCACCTT
UBD -R	GCCGTAATCTGCCATCAT
CXCL1-F	CCGAAGTCATAGCCACAC
CXCL1-R	TTGTCACTGTTCAGCATCT
SOD2-F	TCACCGAGGAGAAGTACC
SOD2-R	TTGATATGACCACCACCATT
CCL20-F	ACACAGACCGTATTCTTCAT
CCL20-R	TACTGAGGAGACGCACAA
B2M-F	ATCCATCCGACATTGAAGT
B2M-R	GGCAGGCATACTCATCTT
TMEM140-F	TCATAGTCCTCGTGATTGTG
TMEM140-R	GTGCTGGTGTCCTCATTC
ARHGEF2-F	TGATGAAGCAGCAAGATGT
ARHGEF2-R	TGAGGAAGCGTGTATGGA

DUSP5-F	GAGTGTTGCGTGGATGTA
DUSP5-R	TCCTCCTCTGCTTGATGTA
NOP16-F	CCTATGTGCTGAATGACCT
NOP16 R	ACCTCCATCTTCCTCTTCT
CSF1-F	GAGGACCAGCAAGTGAAG
CSF1-R	CAGCAAGACCAGGATGAC
TUBA1C-F	TTCCTTCAACACCTTCTTCA
TUBA1C-R	GTTATTGGCAGCATCTTCC
SERPINE1-F	CCACTTCTTCAGGCTGTT
SERPINE1 R	GTTCCAGGATGTCGTAGTAA
CXCL8-F	TCTCTTGGCAGCCTTCCT
CXCL8-R	CTCTCAATCACTCTCAGTTCTT
GLMP-F	TCTGGCAGTGGTAATGGT
GLMP-R	CAAGGAGTATGGAGGATATGG
PVR -F	GAGGATGAAGGCAACTACA
PVR -R	GGCACCAATATCCAGAGG
BIRC3-F	GGAACCGAAGGATAATGCTA
BIRC3-R	GGCTGTCTGATGTGGATAG
RCN1-F	TCTTCAGATCATCACACCTT
RCN1 R	ATCCGCAATATACTCATCCT
GH1-F	AGTTCCTCAGGAGTGTCTT
GH1-R	GTAGTGCGTCATCGTTGT
SAT1-F	CCGTGGATTGGCAAGTTA
SAT1-R	GCAACCTGGCTTAGATTCT
CASP4-F	TGTACTGAACTGGAAGGAAG
CASP4 R	TTGTGCTCTGGTCTGGTA
SBDS-F	CGGTTCATCCTTCCAGTC
SBDS R	CTCATCTCCTTCTTCTACATCT
S100A11-F	TCCAAGACAGAGTTCCTAAG
S100A11-R	GCCACCAATCAGATTAAGAA
LIF-F	ACAACCTGGACAAGCTATG
LIF-R	CACGACTATGCGGTACAG
APOL6-F	ACCTCTACTGCTGTCATCT
APOL6-R	CTGCCTTCTCTTCCTCATC
GDF15-F	GCTACGAGGACCTGCTAA
GDF15-R	GTGCGGACGAAGATTCTG
LAD1-F	AAGGAGGAAGAGGCAGATAT
LAD1-R	CCAGCGAGGAGTCAGATT
VEGFA-F	GGAGGAAGAAGAGAAGGAAG
VEGFA-R	CAGGATGGCTTGAAGATGT
MAPK1IP1L-F	GCACTACCTGAACACTCC
MAPK1IP1L-R	AGATGGCACTGAAGATGG

Supplementary Table 3: The target sequences for lentivirus-based shRNAs used in this paper

Lentivirus	Sequence
LV-TFPI2-RNAi-1	CCTGTGATGCTTTCACCTATA
LV-TFPI2-RNAi-2	CUUGCUGGAGGAUAGAAAATT
LV-CCAR2-RNAi-1	GCCAAAGGAAAGGATCTCTTT
LV-CCAR2-RNAi-2	GCATTGATTTGAGCGGCTGTA
LV-GADD45A-RNAi-1	CTGGAGAGCAGAAGACCGAAA
LV-GADD45A-RNAi-2	GCGAGAACGACAUCAACAUTT
LV-DDX17-RNAi-1	CCAGCAGTTTAGTGGGATA
LV-DDX17-RNAi-2	CTACCAATATGATAGGTTA
LV-BRCC3-RNAi-1	GGCUCAGUGUUUACCAAGATT
LV-BRCC3-RNAi-2	GAAGGACCGUAGUAGAAAUUTT

Supplementary Table 4: The antibodies used in this paper

Antibody	BAND	CAT	WB	IHC	IP	RIP	IF
TFPI2	Abcam	AB186747	1:3000	1:60	3ug		
BRCC3	Proteintech	15391-1-AP	1:3000		3ug		1:100
CCAR2	Proteintech	22638-1-AP	1:3000	1:200	3ug	3ug	1:200
GADD45A	Affinity	DF6622	1:500	1:200			
DDX17	Proteintech	19910-1-AP	1:3000				
GNL1	Proteintech	14078-1-AP	1:1000				
STAT3	Servicebio	GB150001-100	1:1000				
MYC	TransGene	HT101-01	1:3000		3ug		1:100
FLAG	Invitrogen	MA1-91878	1:8000		3ug		1:100
IgG	Proteintech	30000-0-AP			3ug		
Ki67	Proteintech	28074-1-AP		1:10000			
PARP1	Proteintech	13371-1-AP	1:4000	1:300			
γ-H2AX	CST	9718S					1:200
β-actin	Proteintech	81115-1-RR	1:8000				
GAPDH	Proteintech	60004-1-Ig	1:10000				
RAD51	Invitrogen	PA5-27195					1:100
53BP1	CST	4937S					1:200

RNA isolation and RT-qPCR

Total RNA was isolated using the RNAex Pro Reagent (AG21102; ACCURATE BIOTECHNOLOGY). The quantity and density of RNA were detected using a NanoDrop One Microvolume UV-Vis Spectrophotometer (Thermo Scientific, USA) and reverse transcribed using Evo M-MLV RT Premix for qPCR (AG11706, ACCURATE Biotechnology, China). RT-qPCR was performed using Green qPCR Master Mix (AG11701; ACCURATE Biotechnology, China) according to the manufacturer's instructions. The expression of target genes was normalized to that of GAPDH. These assays were performed at least three times, and the relative gene expression was determined using the $2^{-\Delta\Delta Ct}$ method. The primers used for RT-qPCR are listed in **Table S2**.

Plasmids and cell transfection

Lentiviral shRNA vectors for HCC cells, including the non-targeting control, shTFPI2, shBRCC3, shCCAR2, and shGADD45A, were synthesized by GenePharma Co., Ltd. (Shanghai, China). Plasmids for the ectopic expression of indicated genes were synthesized by GenePharma Co., Ltd. (Shanghai, China) and transfected using Lipofectamine 3000 (Invitrogen, Carlsbad, CA, USA). Stably infected cells were selected using puromycin (Selleck, Shanghai, China). The corresponding gene sequences are listed in **Table S3**.

Colony formation assay

Briefly, HCC cells were seeded into a 6-well dish with 2 mL DMEM (10% FBS) at a density of 2000 cells per well. After cell attachment, the cells were treated with DMSO or 4 μM sorafenib. Every three days, culture media was replaced with fresh media comprising DMSO or sorafenib, and colony

formation was examined under a microscope. The colonies were washed with phosphate-buffered saline (PBS) and fixed with 4% paraformaldehyde at room temperature for 1 h. After washing, colonies were stained with 0.1% crystal violet overnight. After air drying, colonies were counted using the ImageJ software (National Institutes of Health, Bethesda, MD).

Western blotting analysis

For western blotting and IP, treated cells were harvested and lysed using a cell lysis buffer (Beyotime, China) containing protease inhibitors (MCE, China) on ice for 30 min, followed by centrifugation to extract the protein supernatant. Protein concentration was quantified using the Pierce BCA Protein Assay Kit (Thermo Scientific, USA). Subsequently, 30 μg of whole protein was mixed with loading buffer and denatured at 100 °C for 10 min. The prepared protein sample was added to the pre-set sodium dodecyl sulfate-polyacrylamide gel electrophoresis lane. The proteins were separated under Tris-glycine electrophoresis conditions and then transferred from the gel to a polyvinylidene difluoride membrane (Millipore, USA). Next, the blots were incubated with the appropriate antibodies. Immobilon Western HRP substrate (Millipore, USA) and a ChemiDoc MP Imaging System (Bio-Rad, USA) were used for blot visualization. The antibodies used are listed in **Table S4**.

Immunohistochemistry

Briefly, the tissue array sections were dehydrated, and the peroxidase activity was quenched. Antigen retrieval was performed in a special pressure cooker for antigen recovery. Tissues were then incubated with primary antibodies overnight at 4 °C and stained with tissue staining kits and 3,3′-diaminobenzidine tetrahydrochloride (DAB). The stained slides were observed, and images were

obtained using a microscope. Three tissue arrays, HLivH160CS02 (80 samples), OD-CT-DgLiv03-002 (31 samples), and OD-CT-DgLiv01 (56 samples), were acquired from Servicebio (Wuhan, China). IHC staining was evaluated using histochemical scoring (H-score), which considers both the strength of staining and the percentage of cells stained at each level of intensity. Staining intensity was assessed and categorized as follows: 0 (no staining), 1 (weak staining), 2 (moderate staining), or 3 (strong staining). The total cell count per field of view and the number of positively stained cells at each intensity level were determined. The proportion of stained cells at each intensity level ranged from 0 to 100%. The H-score was computed using the following formula: H-score = (% of cells stained in intensity category 1 × 1) + (% of cells stained in intensity category 2 × 2) + (% of cells stained in intensity category 3 × 3). The antibodies used are listed in **Table S4**.

TUNEL staining

Cell pretreatment was consistent with the IHC protocol for apoptosis detection using the TUNEL staining method. The treated slides were stained with TUNEL staining solution using a one-step TUNEL Apoptosis Staining Kit (Beyotime, China). Fluorescent images were obtained using a fluorescence microscope.

Apoptosis and cell cycle analyses

Briefly, trypsinization was performed using EDTA-free trypsin, and 1×10^6 cells were collected, washed twice with pre-chilled PBS, and centrifuged to remove the supernatant. For cell cycle analysis, the cells were fixed with 70% ethanol at 4°C for more than 2 h or overnight. The following day, ethanol was removed by centrifugation, and cells were washed twice with PBS to thoroughly remove the remaining

ethanol. Subsequently, the cells were resuspended with RNase buffer and incubated at 37 °C for 30 min. Finally, propidium iodide (PI) staining solution was added, and cells were incubated at 4 °C for 30 min away from light. Red fluorescence at 488 nm was detected using a flow cytometer (NovoCyte Advanteon, China). For cell apoptosis, the RNase-containing resuspension was mixed in proportion with Annexin V-FITC (TransGen, China) and PI staining solution. The cells were suspended in the mixture, gently blown to mix well, and incubated at room temperature in the dark for 15 min. Apoptosis was detected using flow cytometry. Cell DNA content and apoptosis analyses were performed using analysis software, and a map was plotted.

Dual-luciferase reporter assay

Cells were placed in 24-well plates and co-transfected with the appropriate reporter plasmid (pGL3-Basic) and internal reference plasmid (pRL-TK) at 70% confluency using Lipofectamine 3000 (Invitrogen) and maintained for 36 h. Firefly and Renilla luciferase activities were quantified using a dual-luciferase assay kit (Yeasen Biotechnology, Shanghai, China). Renilla luciferase activity was normalized to the firefly luciferase activity. The Glomax multi-detection system was used to measure firefly and Renilla luciferase activities.

Alkaline comet assay

The alkaline comet assay was performed using the Comet Assay Kit (#C2041; Beyotime, China) following the manufacturer's instructions. Briefly, HCC cells were treated with or without 15 μM sorafenib for 24 h, digested with trypsin, and resuspended in ice-cold PBS at a concentration of 2×10^5 cells/mL. Next, 10 μL of cell suspension was mixed with 35 μL preheated comet LMAgarose,

immediately placed on the microscope slides, and then left for 15 min at 4 °C. Slides were carefully submerged in a lysis solution containing DMSO at 4 °C overnight. Following lysis, slides were washed twice with cold PBS (3 min each time), transferred into the pre-cooled alkaline electrophoresis buffer (1 mM EDTA [pH 8.0], 200 mM NaOH [pH 13.0]) for 30 min, and subjected to horizontal gel electrophoresis at 0.75 V/cm for 30 min at 4 °C. Subsequently, excess alkaline electrophoresis buffer was drained, and slides were gently immersed in neutral buffer (0.4 M Tris-HCl, pH 7.5) for 10 min; this step was performed three times. After drying, the slides were stained with 20 μL 1×PI dye for 20 min in the dark at room temperature. Images were obtained using a ZEISS LSM 800 confocal microscope (ZEISS, Germany) at 20× magnification. For each sample, the tail moments of at least 100 cells were measured using OpenComet (v1.3.1, https://cometbio.org), and tail movements were detected.

Homologous recombination (HR) and non-homologous end-joining (NHEJ) reporter assay

For DNA repair reporter analysis, PLCN DSB Repair Reporter (DRR) plasmid (#98,895; Addgene, USA) was transfected into HCC cells with lipo3000, and stable expression cells were selected by G418 (600 μg/mL). Subsequently, HCC NHEJ and HR reporter cells were transfected with TFPI2-overexpressing or Vector, respectively. To induce DSB formation, 0.5 μg of I-SceI and 0.5 μg HR donor (pCAGGS DRR mCherry Donor EF1a BFP, #98,896, Addgene) plasmids were co-transfected into the above reporter cells. After 48 h, cells were subjected to flow cytometry analysis to detect the percentage of GFP+ cells and mCherry+ cells, representing the efficiency of NHEJ or HR repair induced by DNA DSBs. Means were obtained from three independent experiments.

RIP assay

The RIP assay examined the interaction between GADD45A mRNA and CCAR2 protein using the Magna RIP RNA-binding protein immunoprecipitation kit (Millipore, USA). A total of 2×10^7 SNU-449 or MHCC97H cells were harvested, and the cell lysate was incubated with magnetic beads pre-coated with either anti-CCAR2 or anti-IgG antibodies at 4 °C overnight. The RNA-protein complex was then treated with proteinase K at 55 °C for 2 h for protein digestion. Finally, RNA was isolated, purified, and analyzed by RT-qPCR to confirm enrichment.

IP and silver staining

The cells were collected and lysed on ice using the IP lysis buffer (Beyotime, China). Subsequently, the lysates were incubated with protein G magnetic beads (MCE, USA), washed using IP lysis buffer three times for 1 h, and subsequently immunoprecipitated with the indicated antibodies at 4 °C overnight. The lysates were subsequently incubated with protein G Magnetic Beads for 2 h at room temperature, followed by three washes using PBST. The antigen-antibody complexes eluted from the magnetic beads with loading buffer were used for western blot analysis or to detect enriched proteins on polyacrylamide gels using the PierceTM Silver Stain for Mass Spectrometry Kit (Thermo Scientific, USA). Photographs and protein mass spectra were also obtained.
